# Integrative analysis of crotonylation-associated genes reveals prognostic and therapeutic targets in gliomas

**DOI:** 10.3389/fonc.2025.1573997

**Published:** 2025-06-25

**Authors:** Bowen Yin, Zhuoyang Fan, Panpan Yu, Jin Li, Yilin Wang, Minfeng Shu

**Affiliations:** ^1^ Department of Pharmacology, School of Basic Medical Sciences, Shanghai, Medical College, Fudan University, Shanghai, China; ^2^ Key Laboratory of Medical Molecular Virology (Ministry of Education/ National Health Commission/ Chinese Academy of Medical Sciences), Shanghai Frontiers Science Center of Pathogenic Microorganisms and Infection, School of Basic Medical Sciences, Shanghai Medical College, Fudan University, Shanghai, China; ^3^ Department of Interventional Radiology, Zhongshan Hospital, Fudan University, Shanghai, China; ^4^ Department of Hepatic Surgery, Fudan University Shanghai Cancer Center, Shanghai, China; ^5^ Department of Oncology, Shanghai Medical College, Fudan University, Shanghai, China

**Keywords:** glioma, tumor microenvironment, prognosis, machine learning, ChIP-seq, CXCL1

## Abstract

**Background:**

Crotonylation, an emerging epigenetic modification, has been implicated in various biological processes, including tumor progression. However, its role in glioma remains poorly understood. This study aims to investigate the prognostic and therapeutic implications of crotonylation-associated genes in glioma.

**Methods:**

Crotonylation levels were assessed by IHC in glioma tissues of varying grades. Key crotonylation-associated genes were identified and analyzed across five glioma datasets. A prognostic risk score was developed using machine learning algorithms and validated in multiple cohorts. Genomic alterations, immune landscapes, and therapeutic responses were examined in relation to the risk score. Single-cell dataset GSE131928 was analyzed to explore the relationship between the risk score and immune cell infiltration. After crotonate treatment of T98G cells, ChIP-seq and qPCR were performed to investigate the effect of crotonylation on gene expression. Finally, PD-1 and GZMB expression levels were assessed in glioma tissues with varying crotonylation levels.

**Results:**

Crotonylation levels were negatively correlated with glioma grade. Crotonylation-related genes stratified patients into two subtypes with distinct overall survival outcomes. High-risk patients exhibited increased somatic mutations, specific copy number variations, and an immunosuppressive tumor microenvironment. The risk score correlated positively with TIDE scores, indicating resistance to immune checkpoint blockade therapy. Single-cell analysis revealed a positive association between the risk score and TAM infiltration. Candidate therapeutic agents tailored for high- and low-risk groups were identified. ChIP-seq and qPCR demonstrated that reduced crotonylation suppressed *CXCL1* expression and promoted *GZMB* expression in the glioma microenvironment.

**Conclusion:**

Crotonylation-associated genes play a pivotal role in glioma progression and prognosis. The risk score provides a robust tool for patient stratification and treatment guidance, underscoring the importance of crotonylation in glioma biology and its potential as a therapeutic target.

## Introduction

Gliomas, the most common and aggressive primary brain tumors, are characterized by remarkable heterogeneity in their genetic, epigenetic, and immune profiles, posing significant challenges for effective treatment and prognosis (1–3). Among gliomas, glioblastoma (GBM) is the most malignant subtype and remains refractory to current therapeutic modalities (4). The standard treatment, known as the Stupp protocol, combines maximal surgical resection with radiotherapy and concurrent temozolomide (TMZ) chemotherapy (5). Despite these interventions, the prognosis for GBM patients remains dismal, with a median survival of approximately 14–18 months and a high likelihood of recurrence (6). The advent of immunotherapy, which has revolutionized the treatment landscape for cancers such as melanoma and lung cancer (7, 8), has also sparked interest in developing immune-based strategies for glioma. However, challenges such as the immunosuppressive tumor microenvironment and low immunogenicity of gliomas necessitate further exploration of novel biomarkers and therapeutic approaches to improve patient outcomes (9, 10).

Recent studies have uncovered crotonylation, a novel post-translational modification, as a crucial regulatory mechanism in cancer biology (11, 12). This dynamic acylation modification occurs on lysine residues of histones and non-histone proteins, influencing chromatin structure and transcriptional activity (13). Unlike acetylation, crotonylation is associated with transcriptionally active chromatin and is tightly linked to cellular metabolism, particularly the production of Crotonyl-CoA, a key substrate for this modification (14). Crotonylation has been implicated in diverse cellular processes, including DNA repair, cell proliferation, and immune responses, suggesting its potential role in oncogenesis (15, 16). In glioma, while other epigenetic modifications like methylation and acetylation have been extensively studied, the specific contribution of crotonylation to tumor progression, immune regulation, and therapy resistance remains underexplored. Understanding the role of crotonylation-associated genes and their regulatory networks in glioma may uncover novel biomarkers and therapeutic targets, offering new avenues for treatment (17, 18).

We systematically evaluated crotonylation-associated genes in gliomas, focusing on their prognostic and therapeutic significance. By analyzing multi-omics datasets from large glioma cohorts, we constructed a prognostic risk score based on crotonylation-related genes and assessed its relationship with genomic alterations, the immune microenvironment, and therapeutic response. This work highlights the potential of crotonylation-associated genes as biomarkers for glioma stratification and treatment, providing a foundation for future epigenetic studies in gliomas.

## Result

### Identification and prognostic impact of crotonylation-associated gene clusters in glioma

**Figure 1:**
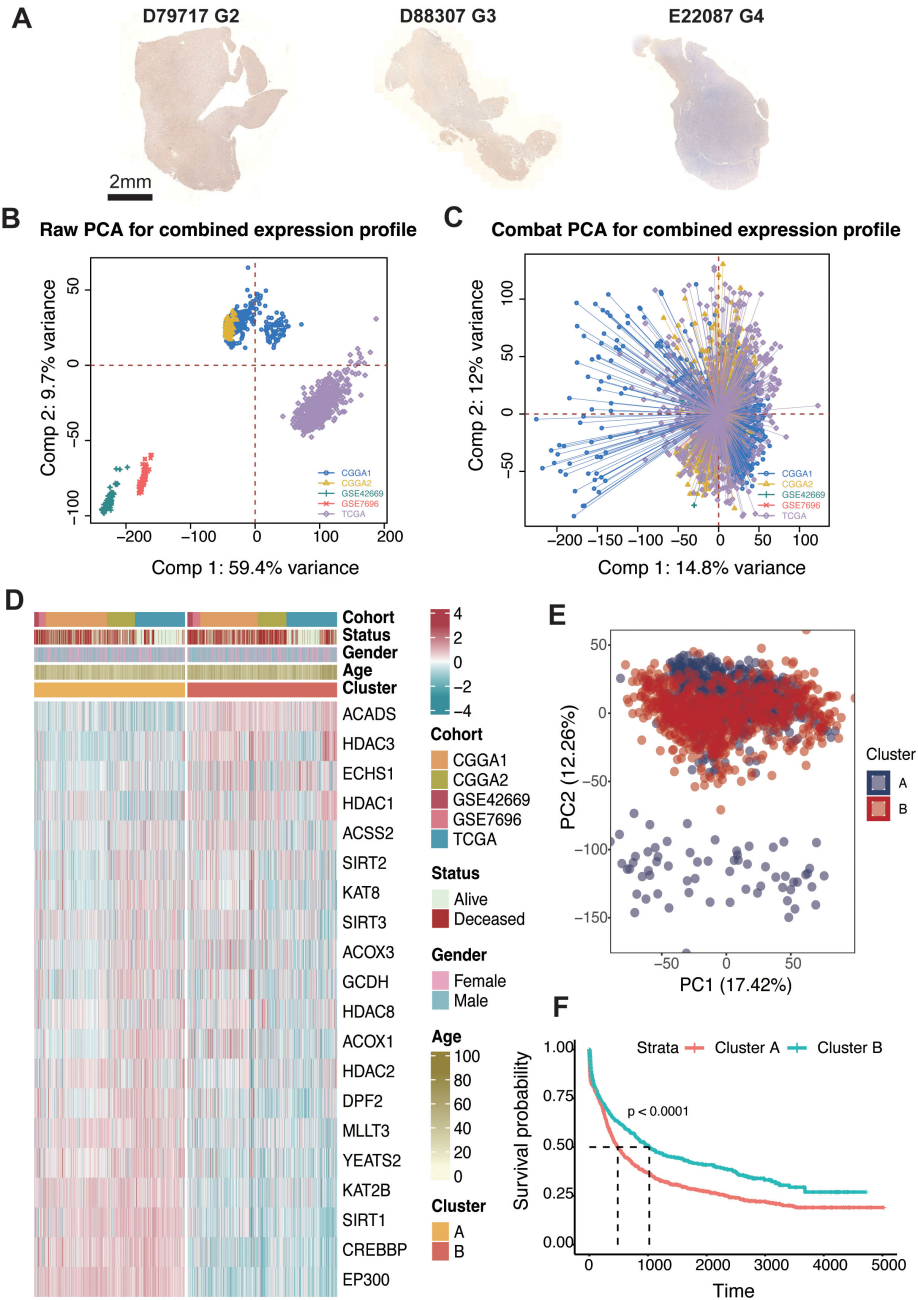
Consensus clustering of crotonylation profiles in glioma. **(A)** IHC staining of Kcr in glioma samples with different grades (G2, G3, G4): D79717, D88307, and E22087. **(B)** PCA of raw expression profiles for the combined dataset. **(C)** PCA after COMBAT adjustment for combined expression profiles. **(D)** Unsupervised clustering of 20 crotonylation-related genes reveals two distinct clusters with patient annotations including cohort source, survival status, gender, and age. **(E)** PCA indicating two distinct clusters within the combined cohorts. **(F)** Kaplan-Meier analysis estimating overall survival differences between Cluster-A and Cluster-B.

We first examined the levels of crotonylation modification in human glioma samples of varying grades (D79717: grade 2; D88307: grade 3; E22087: grade 4). The results revealed a progressive decline in overall crotonylation levels with increasing glioma grade (**Figure 1A**). To identify genes associated with crotonylation, we focused on enzymes involved in the production of Crotonyl-CoA and those catalyzing histone and non-histone crotonylation modifications. Key genes identified “included” *GCDH* and *ECHS1*, both of which play crucial roles in lysine metabolism. Specifically, *GCDH* catalyzes the conversion of glutaryl-CoA to Crotonyl-CoA, while *ECHS1* facilitates the hydration of Crotonyl-CoA to produce 3-hydroxybutyryl-CoA (19–21). Additionally, *ACSS2*, *ACADS*, *ACOX1*, and *ACOX3* are essential in short-chain fatty acid metabolism, where *ACSS2* catalyzes the formation of Crotonyl-CoA from crotonate, and *ACADS*, *ACOX1*, and *ACOX3* catalyze the conversion of butyryl-CoA to Crotonyl-CoA (19, 22, 23). Reported crotonylation “writers” include *EP300*, *CREBBP*, *KAT8*, and *KAT2B*, while “erasers” include *HDAC1*, *HDAC2*, *HDAC3*, *SIRT1*, *SIRT2*, and *SIRT3*. Recognized “readers” of crotonylation modifications include *DPF2*, *MLLT3*, and *YEATS2 (* 11, 12, 24–32) (**Supplementary Table S1**).

To assess the impact of these genes on glioma patient outcomes, we analyzed five glioma datasets: TCGA (GBM + LGG), CGGA1, CGGA2, GSE42669, and GSE7696. Batch effects were minimized across these datasets using the “ComBat” function in the “sva” R package, resulting in reduced inter-dataset variability, thus enabling cohesive analysis (**Figures 1B, C**). To further explore the regulatory role of crotonylation-related genes in relation to glioma patient survival, we applied the ConsensusClusterPlus package in R. Unsupervised clustering revealed several distinct clusters, all with comprehensive survival data. Importantly, two independent clusters, labeled A and B, were identified based on a consensus matrix with k = 2 (**Supplementary Figure S1**; **Figure 1D**). Principal component analysis (PCA) indicated a significant distinction between these two clusters (**Figure 1E**). Survival analysis demonstrated that patients in cluster B were associated with significantly improved prognosis across TCGA (log-rank test, P < 0.001), CGGA1 (log-rank test, P = 0.041), CGGA2 (log-rank test, P < 0.001), GSE7696 (log-rank test, P = 84), GSE42669 (log-rank test, P = 0.011) and the combined cohort (log-rank test, P < 0.001) (**Figure 1E**; **Supplementary Figure S2**).

### Construction of the risk score in glioma

**Figure 2:**
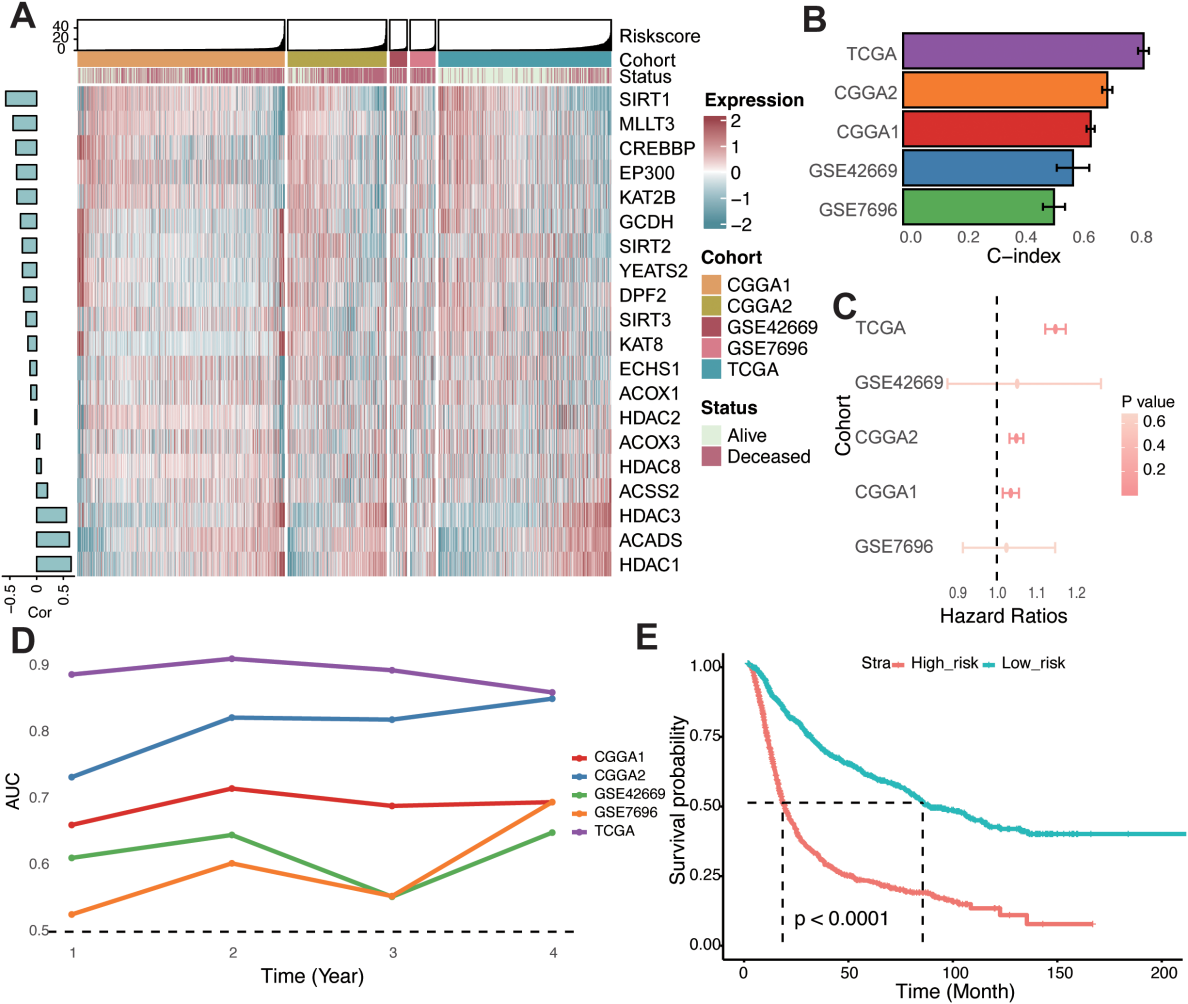
Construction of the risk score in glioma. **(A)** Heatmap showcasing the expression levels of 20 crotonylation-related genes alongside their associated risk scores in combined glioma cohorts. A bar chart to the left displays the correlations between these genes and the risk score. **(B)** Concordance index evaluating the predictive accuracy of the risk score across various cohorts. **(C)** Univariate Cox regression analyses depicting the association between the risk score and survival across different cohorts. **(D)** Time-dependent Area Under the Curve for the risk score across different cohorts. **(E)** Kaplan-Meier survival analysis comparing overall survival rates between low- and high-risk groups in a combined-cohort.

To quantify the relationship between crotonylation-related genes and glioma patient survival, we developed a risk score system tailored to glioma patients. crotonylation-associated genes were incorporated into this system using a variety of machine learning algorithm combinations. Following the approach previously reported (33), we integrated 10 machine learning algorithms—including CoxBoost, Stepwise Cox, Ridge, RSF, GBM, Survival-SVM, Lasso, Enet, plsRcox, and SuperPC—to achieve a risk score with high accuracy and stability across different cohorts. In the TCGA-glioma cohort, over 100 predictive models were constructed, and the average C-index of each model was calculated across four independent validation cohorts (**Supplementary Figure S3**). Among these models, the combination of RSF and StepCox algorithms demonstrated the highest average C-index in the validation cohorts. Genes such as *SIRT*, *MLLT3*, *CREBBP*, *EP300*, *KAT2B*, *GCDH*, *SIRT2*, *YEATS2*, *DPF2*, *SIRT3*, *KAT8*, *ECHS1*, and *ACOX1* were negatively correlated with the risk score, while others exhibited positive correlations (**Figure 2A**).

The C-index of the risk score was also assessed across individual cohorts, where it showed strong predictive performance (**Figure 2B**). Univariate Cox regression analyses confirmed the risk score as an independent prognostic biomarker for overall survival in glioma patients (**Figure 2C**). Furthermore, the time-dependent area under the curve (AUC) values underscored the risk score’s utility as a prognostic indicator in the TCGA, CGGA, and GEO datasets (**Figure 2D**). Based on the median risk score across combined cohorts, glioma patients were categorized into high- and low-risk groups. Kaplan–Meier survival analysis revealed significantly poorer overall survival for patients in the high-risk group across all cohorts (**Figure 2E**; **Supplementary Figure S4**). Collectively, these findings indicate that the risk score is both stable and robust across diverse independent cohorts, making it a valuable tool for glioma prognosis.

### Genomic status of different risk groups

**Figure 3:**
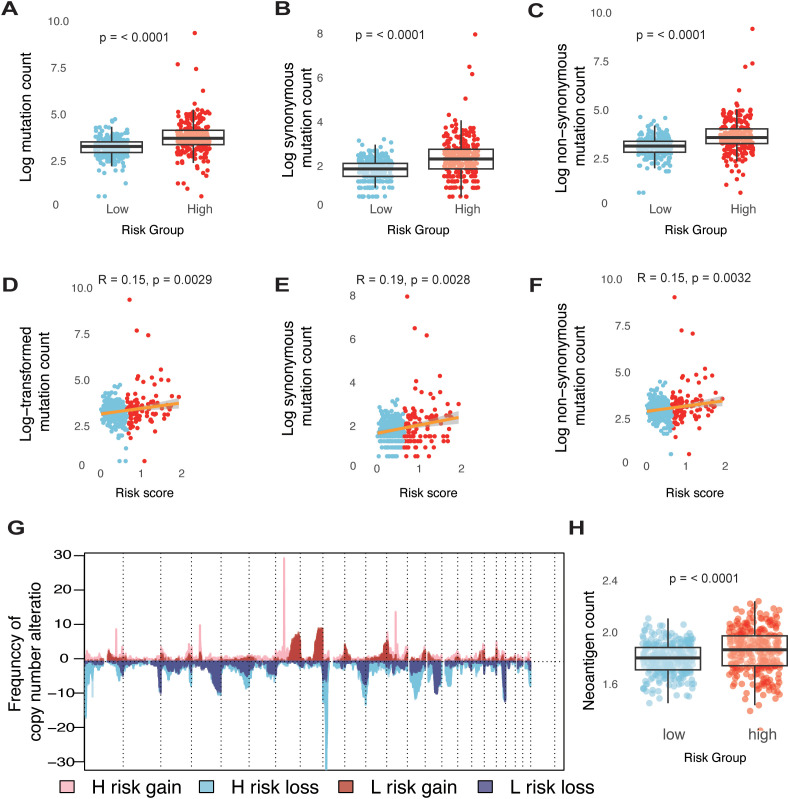
Genomic states of different risk groups in glioma. **(A-C)** Boxplots illustrating comparisons of total mutation counts **(A)**, synonymous mutation counts **(B)**, and non-synonymous mutation counts **(C)** between low- and high-risk groups within the TCGA-glioma cohort. **(D-F)** Analysis of the correlation between risk scores (log transformed) and mutation counts for total mutations **(D)**, synonymous mutations **(E)**, and non-synonymous mutations **(F)** in the TCGA-glioma cohort. **(G)** Overview of copy number variations, showing gains and losses in groups categorized by low and high risk. **(H)** Boxplots detailing the differences in neoantigen counts between low- and high-risk groups in the TCGA-glioma cohort.

To characterize the genomic landscape of different risk groups within the TCGA-glioma dataset, we first examined the frequency of somatic mutations. A positive correlation was identified between the risk score and somatic mutation count, indicating that the high-risk group exhibited a higher frequency of somatic mutations, encompassing both synonymous and non-synonymous types (**Figures 3A–F**). To further explore the relationship between genomic alterations and risk score, we analyzed copy number variations (CNVs) across different risk groups. In the high-risk group, genes on chromosomes 7 and 12 showed a tendency toward amplification, while genes on chromosomes 1, 8, and 13 were more prone to deletion (**Figure 3G**). Genomic instability in gliomas drives somatic mutations and chromosomal copy number variations (CNVs), both of which shape tumor evolution and therapeutic responses. In high-risk gliomas, recurrent CNVs—such as chromosome 7/12 amplifications (*EGFR*, *CDK4*/*MDM2*) and chromosome 1/8/13 deletions (*CHD5*, *DLC1*, *RB1*)—play pivotal roles in promoting proliferation, immune evasion, and DNA repair defects. For instance, *EGFR* amplifications activate PI3K/AKT signaling to fuel tumor growth (3), while *CDK4* gains bypass cell cycle checkpoints, and *RB1* losses impair homologous recombination repair (3, 34, 35). These CNVs synergize with somatic mutations to amplify neoantigen burden, paradoxically creating immunogenic tumors that remain therapy-resistant due to compensatory immunosuppressive mechanisms.

Neoantigens are critical determinants of tumor immunogenicity. In this study, we utilized a neoantigen gene set, defined by mutations capable of generating neoepitopes, to investigate immune response characteristics in glioma. As hypothesized, the high-risk group displayed a significantly higher neoantigen count compared to the low-risk group (**Figure 3H**). These findings indicate that an elevated risk score is linked to an increased mutation burden in glioma tumor cells, potentially contributing to a greater neoantigen load.

### Distinct tumor microenvironment characteristics in high- and low-risk glioma groups

**Figure 4:**
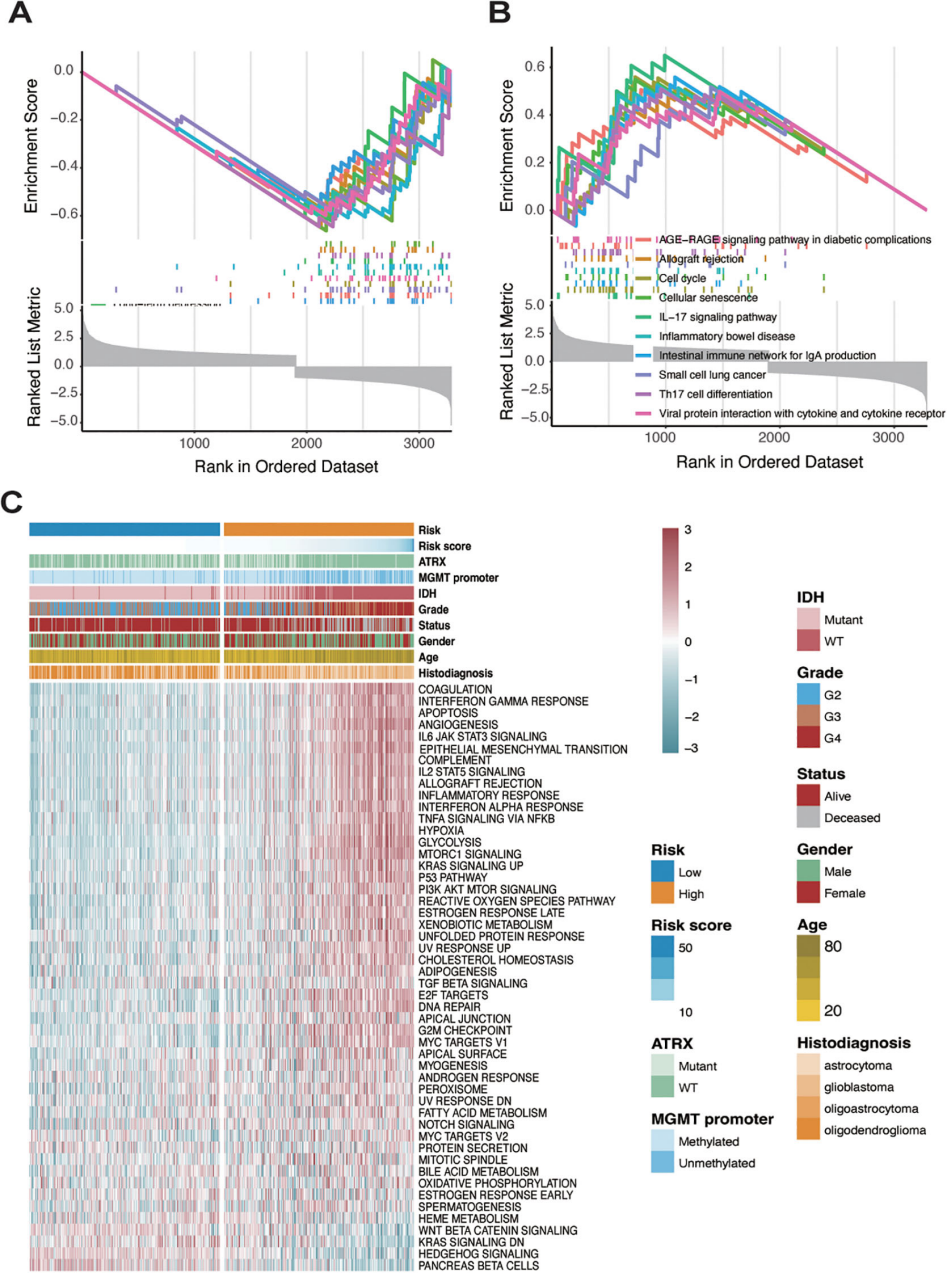
Identification of two distinct patterns in high-risk and low-risk sample groups. **(A)** GSEA analysis showing pathway enrichment of differentially expressed genes analyzed by the limma package in the low-risk group. **(B)** GSEA analysis showing pathway enrichment of differentially expressed genes analyzed by the limma package in the high-risk group. **(C)** Analysis of hallmark gene sets in both low- and high-risk groups.

To characterize the biological features of tumors under the risk classification system, Gene Set Enrichment Analysis was conducted to determine pathways that were associated with differentially expressed genes in high- and low-risk groups. Initially, differential gene expression analysis between the high- and low-risk groups was performed using three R packages: DESeq2, edgeR, and limma (**Supplementary Figure S5**). The results from each package were then analyzed separately to identify the top 10 enriched pathways for each group. In the low-risk group, pathways such as adrenergic signaling in cardiomyocytes, aldosterone synthesis and secretion, cardiac muscle contraction, cortisol synthesis and secretion, long-term depression, and nicotine addiction were predominantly enriched. In contrast, the high-risk group showed enrichment of pathways associated with IL-17 signaling, inflammatory bowel disease, intestinal immune network for IgA production, and viral protein interaction with cytokine and cytokine receptors (**Figures 4A, B**; **Supplementary Figure S6**).

To further investigate the cancer-related pathways underlying these groups, a heatmap was generated using hallmark gene sets from the msigdbr R package, revealing significant expression differences between high- and low-risk groups (**Figure 4C**). In the high-risk group, pathways related to immune and inflammatory responses—such as interferon-gamma response, IL6-JAK-STAT3 signaling, IL2-STAT5 signaling, inflammatory response, and TNF-alpha signaling via NF-kB—were markedly upregulated. In contrast, genes associated with pancreatic beta-cell function, hedgehog signaling, and KRAS signaling were highly expressed in the low-risk group. The methylation status of the MGMT (O6-methylguanine-DNA methyltransferase) promoter is a crucial biomarker in gliomas, particularly in predicting the response to temozolomide (TMZ) chemotherapy. Methylation silences MGMT expression, thereby impairing its ability to repair DNA damage induced by TMZ, leading to better therapeutic outcomes (36, 37). IDH (isocitrate dehydrogenase) mutations, predominantly IDH1 and IDH2, are another critical molecular hallmark, commonly associated with lower-grade gliomas and better prognoses. IDH mutations result in the production of oncometabolites that alter cellular metabolism and epigenetic states, including histone and DNA modifications (38, 39). We observed that patients in the low-risk group were more likely to have a methylated MGMT promoter, while patients in the high-risk group were more frequently characterized by IDH mutations. Moreover, glioblastoma patients, representing high-grade gliomas, were almost exclusively classified into the high-risk group. These findings suggest that the risk scoring system effectively differentiates distinct tumor microenvironments in glioma, highlighting the unique biological characteristics associated with each risk group.

### Immune landscape of different risk groups in glioma

**Figure 5:**
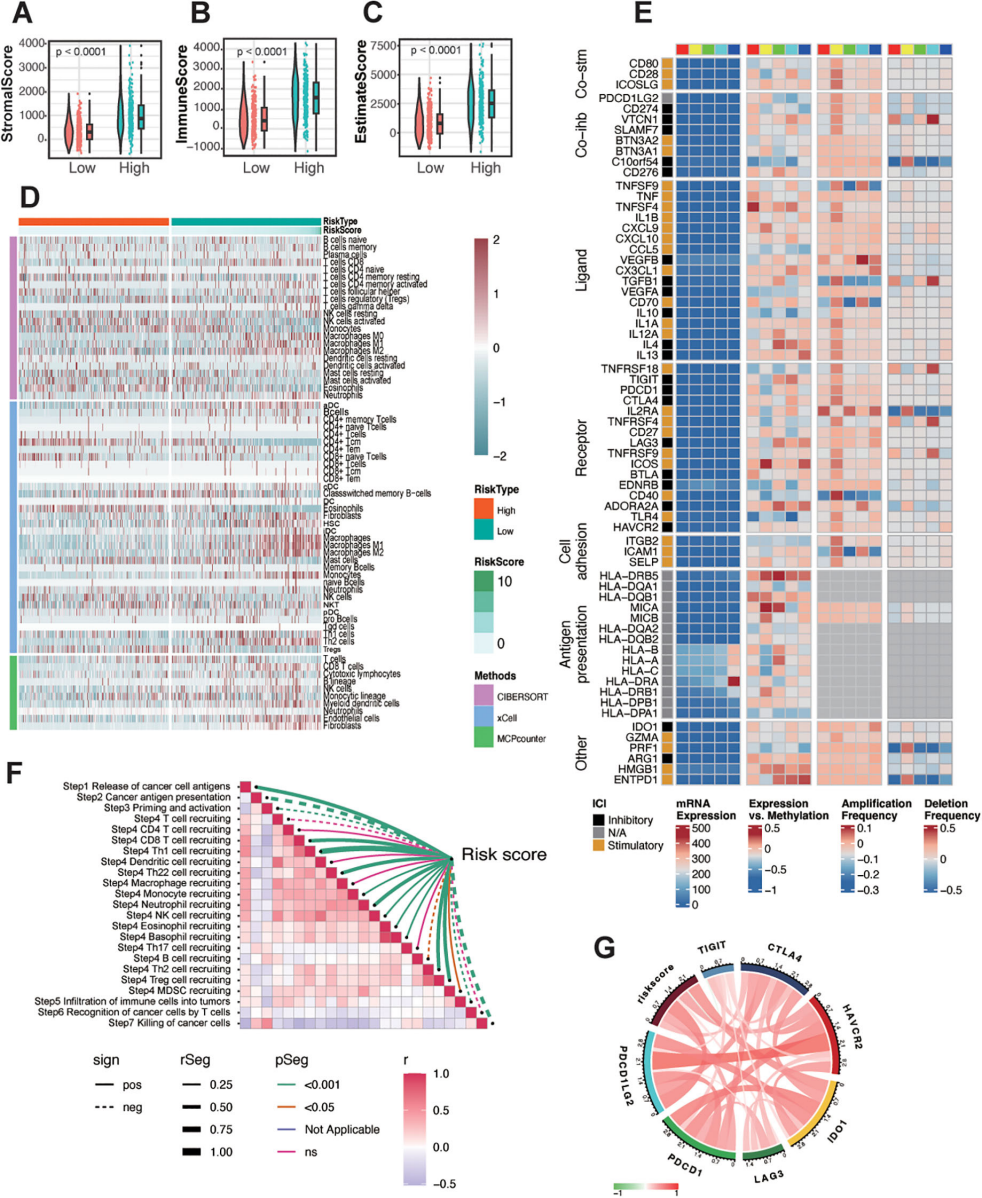
Immune landscape of different risk groups in glioma. **(A-C)** The correlation between the risk score and stromal score **(A)**, immune score **(B)**, and ESTIMATE score **(C)**. **(D)** Heatmap showing the infiltration of immune cells between low- and high-risk groups in the TCGA-glioma cohort. **(E)** Regulation of Immunomodulators, illustrating from left to right: mRNA expression, expression versus methylation, amplification frequency, and deletion frequency (represented as amplifications) for 75 immunomodulatory genes by immune subtype. The colored blocks above, ranging from red, yellow, green, cyan, to blue from left to right, represent the division of the risk score into five equally spaced groups, ordered from smallest to largest. **(F)** Assessment of the risk score correlation with pathways related to anti-tumor immune response steps in TCGA glioma. **(G)** Correlations between the expression of risk score and immune checkpoint markers at mRNA levels.

From the preceding analysis, we observed that the high-risk group exhibited a greater number of neoantigens and significant enrichment in immune response-related pathways. To further characterize the immune status within different risk groups, we employed four distinct methods—ESTIMATE, CIBERSORT, xCELL, and MCPcounter—to evaluate immune cell infiltration. Using the ESTIMATE algorithm, we found that the high-risk group had significantly higher stromal, immune, and ESTIMATE scores compared to the low-risk group (**Figures 5A–C**). Overall, gliomas exhibited limited infiltration of lymphocytes such as CD8+ T cells, CD4+ T cells, and B cells, as well as antigen-presenting cells like dendritic cells, consistent with the generally immunosuppressive nature of gliomas. Comparing the high- and low-risk groups, the high-risk group demonstrated increased infiltration of relatively immunosuppressive cell types, including macrophages (M0, M1, and M2 types), Th2 cells, fibroblasts, monocytes, and neutrophils. Conversely, the low-risk group showed a higher infiltration of NK cells (**Figure 5D**), reflecting distinct immune microenvironments between the two groups.

Given that the risk score was constructed based on crotonylation-associated genes, we further explored the connection between crotonylation and immune-related regulation. Crotonylation, like DNA methylation, acts as an epigenetic regulatory mechanism, with both often working in concert to influence gene expression. Although DNA methylation and histone crotonylation operate on different molecular substrates (DNA versus histones), they are closely interlinked in gene regulation. DNA methylation typically recruits methyl-binding proteins (such as MeCP2) that subsequently recruit HDACs, leading to the removal of histone crotonylation. Since histone crotonylation is associated with an open chromatin structure, its removal results in tighter chromatin conformation, further repressing gene expression (40, 41). To investigate the role of immune-related molecules, we analyzed co-inhibitors, co-stimulators, ligands, receptors, cell adhesion molecules, and antigen presentation factors in glioma. We divided the TCGA-glioma samples into five groups based on their risk scores. Most of these molecules, except for HLA, exhibited relatively low expression levels in glioma. To account for this low expression potentially obscuring their intrinsic trends, we also examined their methylation levels and mutation status, finding that alterations in methylation and mutations were amplified to varying degrees (**Figure 5E**). In particular, IL-13, C10orf54, ENTPD1, TNFSF4, and HLA-DR showed significant correlations with the risk score. Further correlation analysis of expression levels with the risk score revealed a positive association for TNFSF4, HLA-DRA, and ENTPD1(**Supplementary Figure S7**). Additionally, we observed that the risk score negatively correlated with key processes such as T-cell priming and activation, recognition of cancer cells by T cells, and killing of cancer cells (**Figure 5F**). However, it positively correlated with immune checkpoint molecules (**Figure 5G**), suggesting a distinct immune profile linked to the risk score in glioma.

### Predictive value of the risk score in immunotherapy and chemotherapy

To predict immune checkpoint blockade (ICB) response, the TIDE score, a widely recognized predictor, is commonly used to evaluate immune response. The risk score was positively correlated with TIDE and exclusion, while negatively correlated with dysfunction and microsatellite instability (MSI Expr sig) (**Supplementary Figure S8A**). To understand the impact of the risk score on the clinical efficacy of glioma treatments, we analyzed correlations between the risk score and the IC50 of candidate drugs in the Genomics of Drug Sensitivity in Cancer (GDSC) database (**Supplementary Figure S8B**). Over 50 candidate drugs (|Rs| > 0.5) were identified, most of which exhibited a positive correlation between IC50 and the risk score. These drugs targeted IGF-1R inhibitors, CDK4 and CDK6 inhibitors, EGFR-TK inhibitors, and other AMPK inhibitors. Additionally, six drugs exhibited a negative correlation between IC50 and the risk score, including mTOR inhibitors, Lck inhibitors, and Src/Abl inhibitors (**Supplementary Figures S8C, D**).

In summary, these findings suggest that the risk score plays a crucial role in mediating immune response and is associated with drug sensitivity. Thus, the risk score could serve as a potential biomarker for establishing appropriate treatment strategies.

### Exploring the role of CXCL1 and tumor-associated macrophages

**Figure 6:**
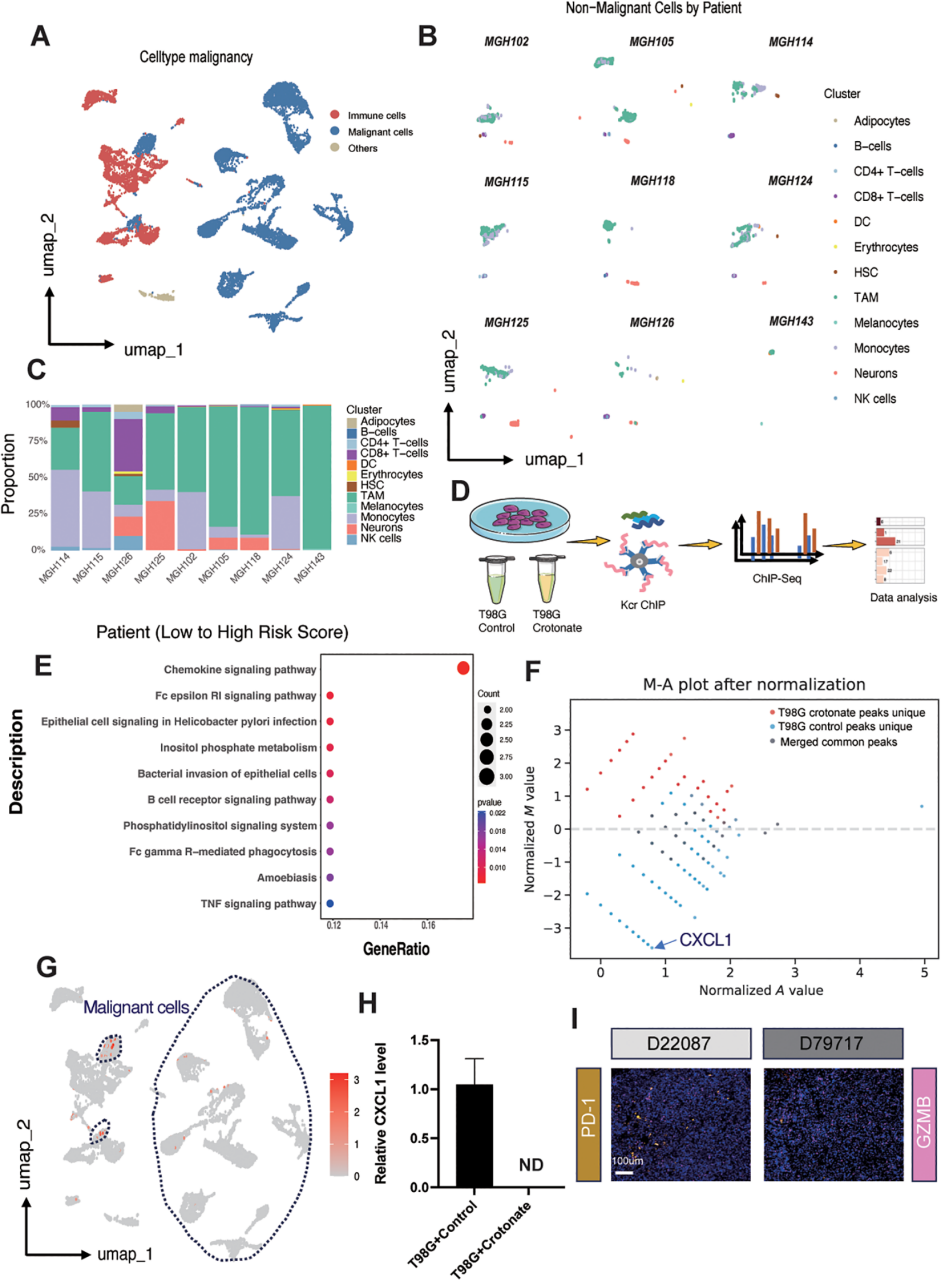
Exploring the role of CXCL1 and tumor-associated macrophages. **(A)** UMAP plot illustrating cell clustering in the single-cell dataset GSE131928. **(B)** UMAP plot displaying immune cell subgroup distributions across different patients. **(C)** Boxplot showing the proportion of different immune cell subgroups ordered by risk score from low to high for each patient in the GSE131928 dataset. **(D)** Schematic representation of the ChIP-seq procedure after crotonate treatment of T98G cells. **(E)** KEGG enrichment analysis highlighting peak differences between crotonate-treated T98G and control T98G cells. **(F)** MA plot depicting the distribution of peaks in control versus crotonate-treated groups. **(G)** UMAP plot demonstrating the relative expression of CXCL1 across different subgroups, with malignant cells circled by dashed lines. **(H)** qPCR bar graph comparing CXCL1 mRNA levels between control and crotonate-treated T98G cells (ND, Not Detected). **(I)** Immunofluorescence staining of PD-1 and GZMB in tissue samples E22087 and D79717. PD-1 is shown in yellow, GZMB in pink, and nuclei are stained with DAPI in blue.

To achieve a higher resolution in detecting the relationship between risk scores and immune cell infiltration, we conducted a series of analyses on the single-cell dataset GSE131928, which includes data from nine glioma patients. First, we clustered the cells based on malignancy, categorizing them into malignant cells, immune cells, and other cells (**Figure 6A**). Then, using SingleR, we further subdivided the non-malignant cell populations and compared the immune cell subgroup distributions across the nine patients, with cluster identities validated by canonical marker genes (**Supplementary Figure S9**). We found that Tumor-Associated Macrophages (TAMs) constituted a relatively large proportion of the cell populations (**Figure 6B**). After calculating the risk scores for each patient, we discovered a positive correlation between the risk score and the infiltration of TAMs (**Figure 6C**), thereby validating our previous results at the single-cell level.

According to reports, crotonylation modification can occur on histone lysine residues, thereby regulating the transcription levels of DNA in those regions (13). To further determine the relationship between TAMs and risk scores, we conducted a series of experiments using the human glioma cell line T98G. Crotonate, a crotonyl-CoA precursor, has been reported to increase intracellular crotonylation modification levels. After treating the cells with crotonate for 48 hours, we performed ChIP-seq analysis using a pan-kcr antibody (**Figure 6D**). KEGG pathway analysis showed that, compared to the crotonate-treated T98G cells, the peaks of chemokine signaling pathway was significantly upregulated in control group (**Figure 6E**), with CXCL1 exhibiting the greatest difference (**Figure 6F**). Umap plots indicated that CXCL1 is relatively high expressed in the malignant cell populations within gliomas (**Figure 6G**). Subsequently, we detected the mRNA levels of CXCL1 using qPCR. The results showed a significant reduction in CXCL1 expression in crotonate-treated T98G cells compared to the control group (**Figure 6H**). CXCL1 is a chemokine that mediates the migration of macrophages to tumor sites through binding with its receptor CXCR2 (42). Tumor cells and surrounding stromal cells secrete CXCL1, creating a chemotactic gradient that attracts monocytes from the circulation into the tumor tissue, where they further differentiate into TAMs. Within the TME, CXCL1 not only participates in the recruitment of macrophages but also influences their polarization (43). TAMs typically exhibit an M2-like phenotype, possessing pro-tumor, pro-angiogenic, and immunosuppressive functions. CXCL1 promotes the polarization of macrophages towards the M2 phenotype by regulating signaling pathways such as PI3K/Akt and STAT3 (44), thereby supporting tumor growth and metastasis. The interaction between CXCL1 and TAMs also involves immunosuppressive mechanisms. M2-TAMs secrete inhibitory cytokines such as IL-10 and express immune checkpoint molecules like PD-L1, which suppress the activity of effector T cells, helping tumors evade immune system surveillance and attack (45). Therefore, we examined the expression levels of PD-1 and GZMB in glioma samples with different levels of crotonylation modification. Consistent with our previous results, PD-1 expression was negatively correlated with crotonylation modification levels, while GZMB expression was positively correlated with crotonylation modification levels (**Figure 6I**).

## Discussion

This study systematically investigates the prognostic and therapeutic significance of crotonylation-associated genes in glioma, revealing their critical role in tumor progression, immune regulation, and patient outcomes. By focusing on the expression and functional impact of crotonylation-related genes across large glioma cohorts, we constructed a robust risk score that effectively stratifies glioma patients into distinct prognostic groups. The results highlight crotonylation as a pivotal epigenetic modification influencing glioma biology, providing a foundation for potential therapeutic interventions.

Crotonylation is a post-translational modification first identified in histones, characterized by the addition of a crotonyl group to lysine residues (13). In the context of cancer, crotonylation has emerged as a significant epigenetic marker influencing tumor biology. Studies have shown that crotonylation plays a role in cancer cell proliferation, invasion, and immune evasion. For instance, crotonylation modifications have been implicated in colorectal cancer and liver cancer, where dysregulated metabolic pathways alter the availability of crotonyl-CoA, affecting chromatin dynamics and gene expression (41, 46, 47). Despite these advancements, research on crotonylation in gliomas remains sparse. Gliomas, particularly glioblastoma multiforme, are known for their heterogeneity and poor prognosis, yet the role of crotonylation in regulating their complex tumor microenvironment and progression is largely unexplored. This paucity of data underscores the need for studies like ours to investigate the potential regulatory mechanisms and therapeutic implications of crotonylation in glioma biology.

The crotonylation-associated risk score in this study was developed using multiple machine learning approaches to identify the optimal combination of prognostic genes (33). Methods such as random forest analysis, and stepwise Cox regression were employed to select genes with the most significant contribution to patient outcomes. Each method offered unique advantages: random forest analysis identified feature importance through ensemble learning, and stepwise Cox regression refined the model to ensure that only the most independent predictors were included. The use of combined machine-learning techniques allowed for a more nuanced and data-driven selection process, capturing complex relationships between gene expression and survival outcomes. Additionally, this combination of methods ensured that the risk score achieved a balance between simplicity and predictive accuracy.

We revealed a significant relationship between the crotonylation-associated risk score and neoantigen burden in glioma patients, suggesting that crotonylation-related genes may influence tumor immunogenicity. High-risk patients exhibited a higher neoantigen load, which is typically associated with increased tumor immunogenicity. However, despite this elevated neoantigen burden, the immune response in these tumors appeared to be suppressed, likely due to the immunosuppressive tumor microenvironment. Gene set enrichment analysis (GSEA) further supported these findings, as immune-related pathways, including those involved in T cell activation, antigen presentation, and cytokine signaling, were significantly enriched in the high-risk group.

Furthermore, our study revealed the predictive value of the risk score in immunotherapy and chemotherapy. High-risk patients exhibited higher TIDE scores, indicating resistance to immune checkpoint blockade (ICB) therapy. This resistance was supported by the positive correlation between the risk score and immune exclusion signatures. Further drug prediction analysis identified potential therapeutic agents with distinct efficacy profiles for high- and low-risk gliomas. These findings emphasize the utility of the risk score in guiding treatment decisions, enabling personalized therapeutic strategies for glioma patients.

Our integrated single-cell and bulk sequencing analyses revealed a significant correlation between tumor-associated macrophages (TAMs) and the crotonylation-associated risk score. TAMs, predominantly polarized into the M2 phenotype, drive tumor progression through multifaceted mechanisms, including immune suppression, hypoxia induction, and metastasis promotion (48). ChIP-seq analysis demonstrated that crotonate treatment globally elevates crotonylation levels but paradoxically reduces peaks at specific loci, such as the chemokine *CXCL1*. We hypothesize: first, although crotonate treatment globally upregulated crotonylation levels, different genes may respond differently to crotonylation modification. Some genes might, in a high crotonylation environment, regulate their crotonylation status through feedback mechanisms or the action of specific de-crotonylases (such as HDACs), leading to a decrease in their crotonylation peaks. Second, crotonylation modifications can affect chromatin openness and its interactions with various epigenetic regulatory proteins. Crotonate treatment may alter the chromatin structure of specific gene regions, reducing their sensitivity to crotonylation modification or changing the binding affinity of transcription factors associated with these modifications, thereby causing a decrease in the crotonylation peaks of these genes. CXCL1, suppressed under high crotonylation conditions, is critical for recruiting monocytes via CXCR2 binding, a process further amplified in hypoxic niches by damage-associated molecular patterns like HMGB1 (49–51). Malignant cells secrete M2-polarizing cytokines (e.g., IL-10, CCL5, CXCL12) to drive their differentiation into immunosuppressive M2 TAMs (52), which in turn secrete IL-10, TGF-β, and HLA-G while recruiting myeloid-derived suppressor cells (MDSCs). MDSCs suppress T-cell function through arginine depletion via arginase-1/iNOS and amplify immunosuppression by producing IL-10, which skews macrophages toward the M2 phenotype (53). M2 TAMs further enhance immune evasion by upregulating PD-L1 and B7-H4, immune checkpoints induced through direct tumor cell contact and correlated with poor prognosis in glioblastoma and hepatocellular carcinoma (54, 55). These checkpoints inhibit cytotoxic T-cell activity, as evidenced by restored CD4+/CD8+ T-cell infiltration following TAM depletion in preclinical models. Importantly, crotonylation is metabolically regulated by lysine-derived crotonyl-CoA, positioning lysine metabolism as a therapeutic lever to modulate epigenetic-immune crosstalk. Dietary or pharmacological interventions targeting lysine intake or HDAC activity could recalibrate crotonylation dynamics, potentially disrupting TAM recruitment and polarization to enhance immunotherapy efficacy in glioma. In parallel, our ongoing development of crotonylation-enhancing therapeutic peptides represents a complementary strategy to directly boost intracellular crotonyl-CoA production through targeted activation of metabolic enzymes. These two approaches, though mechanistically distinct, converge on the common goal of elevating global crotonylation levels – a biochemical modification that appears to exert tumor-suppressive effects through epigenetic reprogramming of both malignant cells and immune components within the glioma ecosystem.

Additionally, our results identified specific immune-related molecules—TNFSF4, HLA-DRA, and ENTPD1—that were significantly correlated with the crotonylation-associated risk score, further linking crotonylation pathways to the regulation of the immune microenvironment in gliomas. Mechanistically, crotonylation-mediated chromatin remodeling alters transcriptional programs, ultimately reshaping the tumor immune landscape and modulating immune exhaustion-related pathways. Among these molecules, ENTPD1 (CD39) stands out due to its well-documented role in tumor immunosuppression. ENTPD1 is an ectonucleotidase that hydrolyzes extracellular ATP into AMP, a precursor of immunosuppressive adenosine. This enzymatic activity contributes to the establishment of an immunosuppressive microenvironment by reducing pro-inflammatory ATP signaling and increasing adenosine-mediated T cell suppression (56–58). Elevated expression of ENTPD1 has been associated with poor prognosis in multiple cancers, including gliomas, where it promotes T cell exhaustion and supports the expansion of regulatory T cells (Tregs). Subsequently, we examined the correlation between Kcr levels and glioma grade in human glioma samples and observed a correlation between Kcr and the expression levels of PD-1 and GZMB in the glioma microenvironment.

Despite these significant findings, our study has some limitations. Gliomas are characterized by profound intra- and intertumoral heterogeneity, which manifests at genetic, epigenetic, and cellular levels. While our risk score effectively stratifies patients into prognostic groups using bulk transcriptomic data, this approach inherently averages subclonal variations. For instance, spatially distinct tumor regions may harbor subclones with divergent crotonylation profiles, potentially leading to underestimation of high-risk subpopulations in bulk analyses. Such heterogeneity could influence the reliability of the risk score in predicting outcomes for patients with mixed molecular subclones. Furthermore, gliomas with heterogeneous IDH mutation status or MGMT promoter methylation—critical molecular subtypes—may exhibit varying crotonylation dynamics. Finer stratification across subtypes could refine the score’s precision in future studies. First, functional studies are needed to elucidate the mechanistic roles of crotonylation-related genes in glioma. Second, the connection between crotonylation and specific immune processes warrants further exploration to fully understand its impact on glioma immunotherapy. Lastly, while the predictive performance of the risk score was robust, additional prospective studies are necessary to confirm its clinical utility. To overcome these limitations, we propose integrating single-cell RNA sequencing and spatial transcriptomics in subsequent research. scRNA-seq would enable resolution of crotonylation-associated gene expression at the single-cell level, identifying rare subclones or immune-stromal interactions masked in bulk data. Spatial techniques could map crotonylation patterns across tumor regions, clarifying their relationship to microenvironmental niches. Additionally, multi-region sampling of tumors in validation cohorts would help quantify regional variability in crotonylation and its prognostic relevance. Finally, validating the risk score within homogeneous molecular subgroups could enhance its clinical applicability.

In conclusion, this study underscores the critical role of crotonylation-associated genes in glioma progression, prognosis, and therapeutic response. The risk score based on these genes provides a valuable tool for stratifying glioma patients and tailoring treatment strategies. Further research into the mechanisms underlying crotonylation and its interplay with the TME may pave the way for novel therapeutic approaches in glioma management.

## Method

### Datasets

RNA sequencing datasets encompassing glioma patients were sourced from multiple databases. For TCGA cohorts, which included Lower Grade Glioma (LGG; WHO grade II–III) and Glioblastoma Multiforme (GBM; WHO grade IV), data were retrieved from the UCSC Xena platform (https://xenabrowser.net/datapage/). Accompanying clinicopathological details were acquired from the cBioPortal (http://www.cbioportal.org/). Additionally, RNA sequencing and clinical data for patients from the Chinese Glioma Genome Atlas (CGGA) were obtained from the CGGA’s official website (https://www.cgga.org.cn). Further, two RNA sequencing datasets (GSE42669, GSE7696 and GSE131928) along with their clinical information were downloaded from the Gene Expression Omnibus (GEO) database. Any patient records lacking survival data were systematically excluded from subsequent survival analyses. Pan-Kcr ChIP-seq data are accessible at GEO under accession codes GSM8532192, GSM8532193, and GSE297117.

### Data processing and mutation analysis

Batch effects across different cohorts were corrected using the “ComBat” algorithm within the “SVA” package. Somatic mutation and copy number alteration (CNA) data for glioma were obtained from TCGA. Additionally, copy number variations associated with glioma were assessed using GISTIC 2.0 through the GenePattern platform.

### Construction of the risk score

To construct the risk score, we implemented a robust methodological framework, evaluating 117 algorithmic combinations to optimize the predictive utility of crotonylation-related genes. This approach incorporated ten distinct algorithms: Lasso, Ridge, Stepwise Cox, CoxBoost, Random Survival Forest (RSF), Elastic Net (Enet), Partial Least Squares Regression for Cox (plsRcox), Supervised Principal Components (SuperPC), Generalized Boosted Regression Modeling (GBM), and Survival Support Vector Machine (Survival-SVM). Each predictive model was subjected to rigorous validation across multiple cohorts, with the Concordance Index (C-index) calculated to quantify predictive accuracy (**Supplementary Table S2**). The final crotonylation risk signature was derived using the RSF and Stepwise Cox algorithms, which yielded the highest average C-index, affirming their superior predictive reliability across the validation cohorts.

### Prediction of response to immunotherapy and chemotherapy

To explore various tumor immune evasion mechanisms, we employed the Tumor Immune Dysfunction and Exclusion (TIDE) algorithm. Chemotherapy response predictions were generated for each sample using the “pRRophetic” package, leveraging a ridge regression model based on cancer drug sensitivity genomics. Additionally, Spearman correlation and differential analyses were performed across risk groups to identify promising therapeutic agents.

### Functional and pathway enrichment analysis

Gene Ontology and Kyoto Encyclopedia of Genes and Genomes analyses were conducted to explore the functions of differentially expressed genes, with significance criteria set at a corrected p-value < 0.05 and an absolute log fold change > 1. Enrichment analysis was performed using the “clusterProfiler” R package (v4.8.3), and results were visualized with the “ggplot2” R package (v3.4.4).

### Estimation of TME cell infiltration

Tumor microenvironment characteristics, including immune, stromal, and ESTIMATE scores for tumor samples, were assessed using the “ESTIMATE” R package. Immune cell infiltration levels within the TME of glioma were quantified using the CIBERSORT, xCell, and MCPcounter algorithms.

### Single-cell data analysis

The single-cell dataset GSE131928 was downloaded from the GEO database. Subsequent quality control, dimensionality reduction, and normalization were performed following the steps outlined on the Seurat website. Cell clustering was conducted using the SingleR package.

### Quantitative real-time PCR

Total RNA of T98G cells was extracted with Trizol reagent (Invi- trogen, USA) following the manufacturer’s instructions.Reverse tran- scription was performed using the 1st strand cDNA Synthesis kit (Yeason, China).quantitative real-time PCR were performed using the Hieff^®^ qPCR SYBR^®^ Green Master Mix (Yeason, China). The relative expression of RNA was calculated according to the 2− ΔΔCT method. The qPCR primers are: H− CXCL1− F: ACAACAATTACGCGCTGCGT; H− CXCL1− R: GTTTCTTAACTATGGGGGATGC; H− ACTIN− F: CACTCTTCCAGCCTTCCTTC; H− ACTIN− R: GTACAGGTCTTTGCGGATGT.

### Immunohistochemistry/immunofluorescence staining

Glioma tissues and paraffin sections were provided by the Department of Neurosurgery, Shanghai Changhai Hospital, Naval Medical University. Tumor tissues were sent to Servicebio for staining analysis.

### Statistical analysis

All statistical analyses were conducted using R software (v4.3.1). Associations between continuous variables were assessed via Spearman’s correlation test, while differences between two groups were evaluated with the Wilcoxon test. Survival differences were analyzed using Kaplan-Meier curves with the log-rank test. A p-value of less than 0.05 was deemed statistically significant.

## Data Availability

The original contributions presented in the study are included in the article/**Supplementary Material**. Further inquiries can be directed to the corresponding authors.

## Supplementary material

Supplementary Figure 1 Delineation of two distinct clusters through crotonylation profiling in glioma. (A) Consensus matrices across combined cohorts for each potential cluster number (k = 2–4), visualized through 1000 iterations of hierarchical clustering. (B) Cumulative Distribution Function (CDF) plot demonstrating the probability distribution based on consensus scores for each k in the combined cohorts. (C) Changes in the area under the CDF curve, expressed relatively. (D) Tracking plot illustrating variations in sample clustering for different k values.

Supplementary Figure 2 Kaplan-Meier analysis for cluster A and cluster B generated after unsupervised clustering of crotonylation-related genes across five datasets. **(A)** TCGA, **(B)** CGGA1, **(C)** CGGA2, **(D)** GSE7696, **(E)** GSE42669.

Supplementary Figure 3 Illustration of the machine learning-based integrative procedure. This figure demonstrates a comprehensive evaluation involving a total of 117 predictive models, with subsequent calculation of the Concordance Index for each model across all validation datasets.

Supplementary Figure 4 Kaplan-Meier analysis for high- and low-risk groups across five datasets. **(A)** TCGA, **(B)** CGGA1, **(C)** CGGA2, **(D)** GSE7696, **(E)** GSE42669.

Supplementary Figure 5 Differential gene analysis between high-risk and low-risk samples in glioma. (A) Heatmaps displaying differential gene expression between high-risk and low-risk samples analyzed using the DESeq2 (left), edgeR (center), and limma (right) packages in R. (B) Volcano plots illustrating upregulated (red) and downregulated (blue) genes in high-risk versus low-risk samples, as identified by DESeq2 (left), edgeR (center), and limma (right) analyses.

Supplementary Figure 6 Pathway enrichment analyses in low-risk and high-risk Groups. **(A, B)** GSEA analysis showing pathway enrichment of differentially expressed genes analyzed by the DESeq2 package in the low-risk group **(A)** and high-risk group **(B)**. **(C, D)** GSEA analysis showing pathway enrichment of differentially expressed genes analyzed by the edgeR package in the low-risk group **(C)** and high-risk group **(D)**.

Supplementary Figure 7 Correlation analysis between the risk score and mRNA expression levels. **(A)** TNFSF4, **(B)** IL13, **(C)** C0orf54, **(D)** HLA-DRA, and **(E)** ENTPD1.

Supplementary Figure 8 Predictive value of the risk score in immunotherapy and chemotherapy. **(A)** The correlations between the risk score with TIDE score, dysfunction score, exclusion score, and MSI expression signature. **(B)** The relation between the IC50 of candidate drugs and risk scores. **(C, D)** Boxplots showing the estimated higher IC50 values of drugs in the high-risk group **(C)** and low-risk group **(D)**.

Supplementary Figure 9 Validation of SingleR cell type annotation accuracy using lineage-specific marker genes.

Supplementary Figure 10 Proposed mechanism of crotonylation-mediated regulation of macrophage infiltration and polarization in gliomas. Elevated intracellular crotonyl-CoA levels in glioma cells enhance global crotonylation, including histone crotonylation modifications. This epigenetic alteration induces chromatin structural remodeling, leading to transcriptional repression of the chemokine *CXCL1*. Reduced CXCL1 secretion into the tumor microenvironment diminishes monocyte recruitment via CXCR2 signaling, thereby suppressing tumor-associated macrophage (TAM) infiltration.

## References

[1] Louis DN Perry A Reifenberger G von Deimling A Figarella-Branger D Cavenee WK. The 2016 World Health Organization classification of tumors of the central nervous system: a summary. Acta Neuropathol (Berl). (2016) 131:803–20. doi: 10.1007/s00401-016-1545-1 27157931

[2] Verhaak RGW Hoadley KA Purdom E Wang V Qi Y Wilkerson MD. An integrated genomic analysis identifies clinically relevant subtypes of glioblastoma characterized by abnormalities in PDGFRA, IDH1, EGFR and NF1. Cancer Cell. (2010) 17:98. doi: 10.1016/j.ccr.2009.12.020 20129251 PMC2818769

[3] Brennan CW Verhaak RGW McKenna A Campos B Noushmehr H Salama SR. The somatic genomic landscape of glioblastoma. Cell. (2013) 155:462–77. doi: 10.1016/j.cell.2013.09.034 PMC3910500 24120142

[4] Omuro A DeAngelis LM. Glioblastoma and other Malignant gliomas: a clinical review. JAMA. (2013) 310(17):1842–50. doi: 10.1001/jama.2013.280319 24193082

[5] Stupp R Hegi ME Mason WP van den Bent MJ Taphoorn MJB Janzer RC. Effects of radiotherapy with concomitant and adjuvant temozolomide versus radiotherapy alone on survival in glioblastoma in a randomised phase III study: 5-year analysis of the EORTC-NCIC trial. Lancet Oncol. (2009) 10:459–66. doi: 10.1016/S1470-2045(09)70025-7 19269895

[6] Weller M Wen PY Chang SM Dirven L Lim M Monje M. Glioma. Nat Rev Dis Primer. (2024) 10:33. doi: 10.1038/s41572-024-00516-y 38724526

[7] Wolchok JD Chiarion-Sileni V Gonzalez R Rutkowski P Grob J-J Cowey CL. Overall survival with combined nivolumab and ipilimumab in advanced melanoma. N Engl J Med. (2017) 377:1345–56. doi: 10.1056/NEJMoa1709684 PMC5706778 28889792

[8] Gandhi L Rodríguez-Abreu D Gadgeel S Esteban E Felip E De Angelis F. Pembrolizumab plus chemotherapy in metastatic non-small-cell lung cancer. N Engl J Med. (2018) 378(22):2078–92. doi: 10.1056/NEJMoa1801005 29658856

[9] Hambardzumyan D Gutmann DH Kettenmann H. The role of microglia and macrophages in glioma maintenance and progression. Nat Neurosci. (2016) 19(1):20–7. doi: 10.1038/nn.4185 PMC4876023 26713745

[10] Quail DF Joyce JA. The microenvironmental landscape of brain tumors. Cancer Cell. (2017) 31:326–41. doi: 10.1016/j.ccell.2017.02.009 PMC5424263 28292436

[11] Sabari BR Tang Z Huang H Yong-Gonzalez V Molina H Kong HE. Intracellular crotonyl-CoA stimulates transcription through p300-catalyzed histone crotonylation. Mol Cell. (2015) 58:203–15. doi: 10.1016/j.molcel.2015.02.029 PMC4501262 25818647

[12] Zhao D Guan H Zhao S Mi W Wen H Li Y. YEATS2 is a selective histone crotonylation reader. Cell Res. (2016) 26:629–32. doi: 10.1038/cr.2016.49 PMC4856769 27103431

[13] Tan M Luo H Lee S Jin F Yang JS Montellier E. Identification of 67 histone marks and histone lysine crotonylation as a new type of histone modification. Cell. (2011) 146:1016–28. doi: 10.1016/j.cell.2011.08.008 PMC3176443 21925322

[14] Wei W Mao A Tang B Zeng Q Gao S Liu X. Large-scale identification of protein crotonylation reveals its role in multiple cellular functions. J Proteome Res. (2017) 16:1743–52. doi: 10.1021/acs.jproteome.7b00012 28234478

[15] Liu N Konuma T Sharma R Wang D Zhao N Cao L. Histone H3 lysine 27 crotonylation mediates gene transcriptional repression in chromatin. Mol Cell. (2023) 83(13):2206–21.e11. doi: 10.1016/j.molcel.2023.05.022 PMC11138481 37311463

[16] Liu S Xue C Fang Y Chen G Peng X Zhou Y. Global involvement of lysine crotonylation in protein modification and transcription regulation in rice. Mol Cell Proteomics MCP. (2018) 17(10):1922–36. doi: 10.1074/mcp.RA118.000640 PMC6166680 30021883

[17] Narita T Weinert BT Choudhary C. Functions and mechanisms of non-histone protein acetylation. Nat Rev Mol Cell Biol. (2019) 20:156–74. doi: 10.1038/s41580-018-0081-3 30467427

[18] Huang H Wang DL Zhao Y. Quantitative crotonylome analysis expands the roles of p300 in the regulation of lysine crotonylation pathway. Proteomics. 18(15):e1700230. doi: 10.1002/pmic.201700230 PMC6420807 29932303

[19] Yuan H Wu X Wu Q Chatoff A Megill E Gao J. Lysine catabolism reprograms tumour immunity through histone crotonylation. Nature. (2023) 617:818–26. doi: 10.1038/s41586-023-06061-0 PMC11089809 37198486

[20] Lao Y Cui X Xu Z Yan H Zhang Z Zhang Z. Glutaryl-CoA dehydrogenase suppresses tumor progression and shapes an anti-tumor microenvironment in hepatocellular carcinoma. J Hepatol. (2024) 81:847–61. doi: 10.1016/j.jhep.2024.05.034 38825017

[21] Guo Z Zhang Y Wang H Liao L Ma L Zhao Y. Hypoxia-induced downregulation of PGK1 crotonylation promotes tumorigenesis by coordinating glycolysis and the TCA cycle. Nat Commun. (2024) 15:6915. doi: 10.1038/s41467-024-51232-w 39134530 PMC11319824

[22] Comerford SA Huang Z Du X Wang Y Cai L Witkiewicz AK. Acetate dependence of tumors. Cell. (2014) 159:1591–602. doi: 10.1016/j.cell.2014.11.020 PMC4272450 25525877

[23] Zhang Y Chen Y Zhang Z Tao X Xu S Zhang X. Acox2 is a regulator of lysine crotonylation that mediates hepatic metabolic homeostasis in mice. Cell Death Dis. (2022) 13:279. doi: 10.1038/s41419-022-04725-9 35351852 PMC8964741

[24] Xu W Wan J Zhan J Li X He H Shi Z. Global profiling of crotonylation on non-histone proteins. Cell Res. (2017) 27:946–9. doi: 10.1038/cr.2017.60 PMC5518986 28429772

[25] Liu X Wei W Liu Y Yang X Wu J Zhang Y. MOF as an evolutionarily conserved histone crotonyltransferase and transcriptional activation by histone acetyltransferase-deficient and crotonyltransferase-competent CBP/p300. Cell Discov. (2017) 3:17016. doi: 10.1038/celldisc.2017.16 28580166 PMC5441097

[26] Madsen AS Olsen CA. Profiling of substrates for zinc-dependent lysine deacylase enzymes: HDAC3 exhibits decrotonylase activity in *vitro.* Angew. Chem Int Ed Engl. (2012) 51:9083–7. doi: 10.1002/anie.201203754 22890609

[27] Feldman JL Baeza J Denu JM. Activation of the protein deacetylase SIRT6 by long-chain fatty acids and widespread deacylation by mammalian sirtuins. J Biol Chem. (2013) 288:31350–6. doi: 10.1074/jbc.C113.511261 PMC3829447 24052263

[28] Bao X Wang Y Li X Li X-M Liu Z Yang T. Identification of “erasers” for lysine crotonylated histone marks using a chemical proteomics approach. eLife. (2014) 3:e02999. doi: 10.7554/eLife.02999 25369635 PMC4358366

[29] Wei W Liu X Chen J Gao S Lu L Zhang H. Class I histone deacetylases are major histone decrotonylases: evidence for critical and broad function of histone crotonylation in transcription. Cell Res. (2017) 27:898–915. doi: 10.1038/cr.2017.68 28497810 PMC5518989

[30] Kelly RDW Chandru A Watson PJ Song Y Blades M Robertson NS. Histone deacetylase (HDAC) 1 and 2 complexes regulate both histone acetylation and crotonylation in *vivo* . Sci Rep. (2018) 8:14690. doi: 10.1038/s41598-018-32927-9 30279482 PMC6168483

[31] Xiong X Panchenko T Yang S Zhao S Yan P Zhang W. Selective recognition of histone crotonylation by double PHD fingers of MOZ and DPF2. Nat. Chem Biol. (2016) 12:1111–8. doi: 10.1038/nchembio.2218 PMC5253430 27775714

[32] Zhang Q Zeng L Zhao C Ju Y Konuma T Zhou M-M. Structural insights into histone crotonyl-lysine recognition by the AF9 YEATS domain. Struct Lond Engl 1993. (2016) 24:1606–12. doi: 10.1016/j.str.2016.05.023 PMC5014688 27545619

[33] Liu Z Liu L Weng S Guo C Dang Q Xu H. Machine learning-based integration develops an immune-derived lncRNA signature for improving outcomes in colorectal cancer. Nat Commun. (2022) 13:816. doi: 10.1038/s41467-022-28421-6 35145098 PMC8831564

[34] Cancer Genome Atlas Research Network Brat DJ Verhaak RGW Aldape KD Yung WKA Salama SR. Comprehensive, integrative genomic analysis of diffuse lower-grade gliomas. N Engl J Med. (2015) 372:2481–98. doi: 10.1056/NEJMoa1402121 PMC4530011 26061751

[35] Wang Q Hu B Hu X Kim H Squatrito M Scarpace L. Tumor evolution of glioma-intrinsic gene expression subtypes associates with immunological changes in the microenvironment. Cancer Cell. (2017) 32:42–56.e6. doi: 10.1016/j.ccell.2017.06.003 28697342 PMC5599156

[36] Hegi ME Diserens A-C Gorlia T Hamou M-F de Tribolet N Weller M. MGMT gene silencing and benefit from temozolomide in glioblastoma. N Engl J Med. (2005) 352:997–1003. doi: 10.1056/NEJMoa043331 15758010

[37] Stupp R Taillibert S Kanner AA Kesari S Steinberg DM Toms SA. Maintenance therapy with tumor-treating fields plus temozolomide vs temozolomide alone for glioblastoma: A randomized clinical trial. JAMA. (2015) 314(23):2535–43. doi: 10.1001/jama.2015.16669 26670971

[38] Dang L White DW Gross S Bennett BD Bittinger MA Driggers EM. Cancer-associated IDH1 mutations produce 2-hydroxyglutarate. Nature. (2009) 462:739–44. doi: 10.1038/nature08617 PMC2818760 19935646

[39] Lu C Ward PS Kapoor GS Rohle D Turcan S Abdel-Wahab O. IDH mutation impairs histone demethylation and results in a block to cell differentiation. Nature. (2012) 483:474–8. doi: 10.1038/nature10860 PMC3478770 22343901

[40] Li Y Sabari BR Panchenko T Wen H Zhao D Guan H. Molecular coupling of histone crotonylation and active transcription by AF9 YEATS domain. Mol Cell. (2016) 62:181–93. doi: 10.1016/j.molcel.2016.03.028 PMC4841940 27105114

[41] Sabari BR Zhang D Allis CD Zhao Y. Metabolic regulation of gene expression through histone acylations. Nat Rev Mol Cell Biol. (2017) 18:90–101. doi: 10.1038/nrm.2016.140 27924077 PMC5320945

[42] Wang S Bai J Zhang YL Lin QY Han X Qu WK. CXCL1-CXCR2 signalling mediates hypertensive retinopathy by inducing macrophage infiltration. Redox Biol. (2022) 56:102438. doi: 10.1016/j.redox.2022.102438 35981418 PMC9418605

[43] Wu H Jiang N Li J Jin Q Jin J Guo J. Tumor cell SPTBN1 inhibits M2 polarization of macrophages by suppressing CXCL1 expression. J Cell Physiol. (2024) 239:97–111. doi: 10.1002/jcp.31146 37921259

[44] Xu X-S Feng Z-H Cao D Wu H Wang M-H Li J-Z. SCARF1 promotes M2 polarization of Kupffer cells via calcium-dependent PI3K-AKT-STAT3 signalling to improve liver transplantation. Cell Prolif. (2021) 54:e13022. doi: 10.1111/cpr.13022 33686740 PMC8016636

[45] Goswami KK Bose A Baral R. Macrophages in tumor: An inflammatory perspective. Clin Immunol Orlando Fla. (2021) 232:108875. doi: 10.1016/j.clim.2021.108875 34740843

[46] Liao M Sun X Zheng W Wu M Wang Y Yao J. LINC00922 decoys SIRT3 to facilitate the metastasis of colorectal cancer through up-regulation the H3K27 crotonylation of ETS1 promoter. Mol Cancer. (2023) 22(1):163. doi: 10.1186/s12943-023-01859-y 37789393 PMC10548613

[47] Zhang D Tang J Xu Y Huang X Wang Y Jin X. Global crotonylome reveals hypoxia-mediated lamin A crotonylation regulated by HDAC6 in liver cancer. Cell Death Dis. (2022) 13:717. doi: 10.1038/s41419-022-05165-1 35977926 PMC9385620

[48] Mosser DM Edwards JP. Exploring the full spectrum of macrophage activation. Nat Rev Immunol. (2008) 8(12):958–69. doi: 10.1038/nri2448 PMC2724991 19029990

[49] Liu Y Yan W Tohme S Chen M Fu Y Tian D. Hypoxia induced HMGB1 and mitochondrial DNA interactions mediate tumor growth in hepatocellular carcinoma through Toll-like receptor 9. J Hepatol. (2015) 63(1):114–21. doi: 10.1016/j.jhep.2015.02.009 PMC4475488 25681553

[50] Gebhardt C Riehl A Durchdewald M Németh J Fürstenberger G Müller-Decker K. RAGE signaling sustains inflammation and promotes tumor development. J Exp Med. (2008) 205(2):275–85. doi: 10.1084/jem.20070679 PMC2271015 18208974

[51] Mittal D Saccheri F Vénéreau E Pusterla T Bianchi ME Rescigno M. TLR4-mediated skin carcinogenesis is dependent on immune and radioresistant cells. EMBO J. (2010) 29(13):2242–52. doi: 10.1038/emboj.2010.94 PMC2905255 20526283

[52] Zhang M He Y Sun X Li Q Wang W Zhao A. A high M1/M2 ratio of tumor-associated macrophages is associated with extended survival in ovarian cancer patients. J Ovarian Res. (2014) 7:19. doi: 10.1186/1757-2215-7-19 24507759 PMC3939626

[53] Parker KH Sinha P Horn LA Clements VK Yang H Li J. HMGB1 enhances immune suppression by facilitating the differentiation and suppressive activity of myeloid-derived suppressor cells. Cancer Res. (2014) 74(20):5723–33. doi: 10.1158/0008-5472.CAN-13-2347 PMC4199911 25164013

[54] Jeong H Kim S Hong B-J Lee C-J Kim Y-E Bok S. Tumor-associated macrophages enhance tumor hypoxia and aerobic glycolysis. Cancer Res. (2019) 79:795–806. doi: 10.1158/0008-5472.CAN-18-2545 30610087

[55] Bloch O Crane CA Kaur R Safaee M Rutkowski MJ Parsa AT. Gliomas promote immunosuppression through induction of B7-H1 expression in tumor-associated macrophages. Clin. Cancer Res. Off. J. Am. Assoc. Cancer Res. (2013) 19:3165–75. doi: 10.1158/1078-0432.CCR-12-3314 PMC3742575 23613317

[56] Kazemi MH Raoofi Mohseni S Hojjat-Farsangi M Anvari E Ghalamfarsa G Mohammadi H. Adenosine and adenosine receptors in the immunopathogenesis and treatment of cancer. J Cell Physiol. (2018) 233:2032–57. doi: 10.1002/jcp.25873 28233320

[57] Vijayan D Young A Teng MWL Smyth MJ. Targeting immunosuppressive adenosine in cancer. Nat Rev Cancer. (2017) 17:765. doi: 10.1038/nrc.2017.110 29162946

[58] Allard D Allard B Gaudreau P-O Chrobak P Stagg J. CD73-adenosine: a next-generation target in immuno-oncology. Immunotherapy. (2016) 8:145–63. doi: 10.2217/imt.15.106 26808918

