# Supplementary material for: Integrative analysis of crotonylation-associated genes reveals prognostic and therapeutic targets in gliomas
Source: Front Oncol. 2025 Jun 25;15:1573997. doi: 10.3389/fonc.2025.1573997 (PMC12237899; doi:10.3389/fonc.2025.1573997)
Supplement: Supplementary file 1 [file Table1.docx]

| Dataset Name | Type | Sample size/Patients | Platform |
| --- | --- | --- | --- |
| GSE42669 | Expression profiling by array | 58 | Affymetrix Human Gene 1.0 ST Array |
| GSE7696 | Expression profiling by array | 84 | Affymetrix Human Genome U133 Plus 2.0 Array |
| GSE131928 | Single sell sequencing | 9 (13553 cells) | 10x genomics |

Table S1. Summary GEO datasets
