# Supplementary material for: Integrative analysis of crotonylation-associated genes reveals prognostic and therapeutic targets in gliomas
Source: Front Oncol. 2025 Jun 25;15:1573997. doi: 10.3389/fonc.2025.1573997 (PMC12237899; doi:10.3389/fonc.2025.1573997)
Supplement: Supplementary file 2 [file Table2.docx]

Table S2. Summary of Machine Learning Parameters

| **Algorithm** | **Key Parameters** | **Tuning Method** | **Tuning Range/Options** | **Final Value** |
| --- | --- | --- | --- | --- |
| **RSF** | ntree (number of trees) | Fixed value | 1000 | 1000 |
|  | nodesize (minimum node size) | Grid search | 5, 10, 20 | 10 |
|  | splitrule | Fixed value | "logrank" | "logrank" |
| **CoxBoost** | penalty (regularization parameter) | Optimized via optimCoxBoostPenalty | Start = 500, iteratively adjusted | Derived from optimization |
|  | stepno (number of boosting steps) | 10-fold cross-validation | 1–500 steps | Optimal step from CV |
| **Elastic Net** | α (mixing parameter) | Grid search | 0.1–0.9 (step size 0.1) | Optimal α via CV (e.g., 0.5) |
|  | λ (regularization strength) | 10-fold cross-validation | Automatically determined | lambda.min |
| **GBM** | n.trees (number of trees) | 10-fold cross-validation | Initial: 10,000; pruned to optimal | Optimal trees (e.g., 1500) |
|  | interaction.depth (tree complexity) | Fixed value | 3 | 3 |
|  | shrinkage (learning rate) | Fixed value | 0.001 | 0.001 |
| **Lasso** | α | Fixed value | 1 | 1 |
|  | λ | 10-fold cross-validation | Automatically determined | lambda.min |
| **Ridge** | α | Fixed value | 0 | 0 |
|  | λ | 10-fold cross-validation | Automatically determined | lambda.min |
| **StepCox** | direction (variable selection) | Tested all options | "forward," "backward," "both" | Best-performing direction |
| **SuperPC** | threshold (feature inclusion cutoff) | Cross-validation | 20 thresholds tested | Optimal threshold from CV |
|  | n.components (principal components) | Fixed value | 1 | 1 |
| **plsRcox** | nt (number of components) | 10-fold cross-validation | 1–10 components | Optimal nt from CV |
| **survival-SVM** | gamma.mu (kernel parameter) | Default value | 1 (no tuning) | 1 |
