# Supplementary figures and images for: Integrative analysis of crotonylation-associated genes reveals prognostic and therapeutic targets in gliomas
Source: Front Oncol. 2025 Jun 25;15:1573997. doi: 10.3389/fonc.2025.1573997 (PMC12237899; doi:10.3389/fonc.2025.1573997)

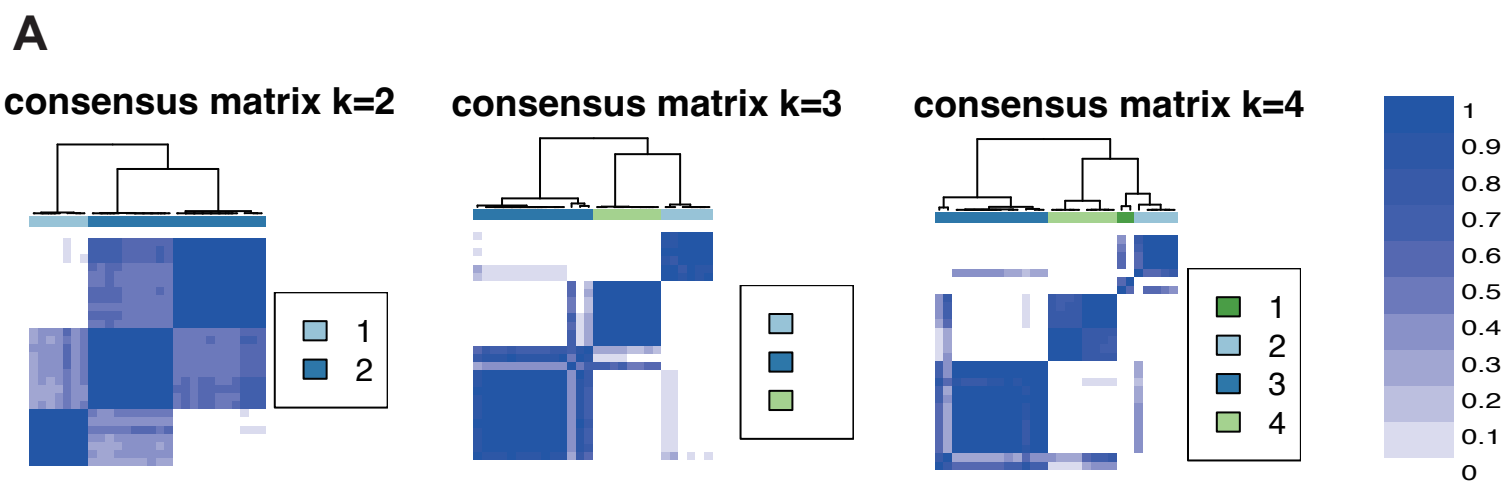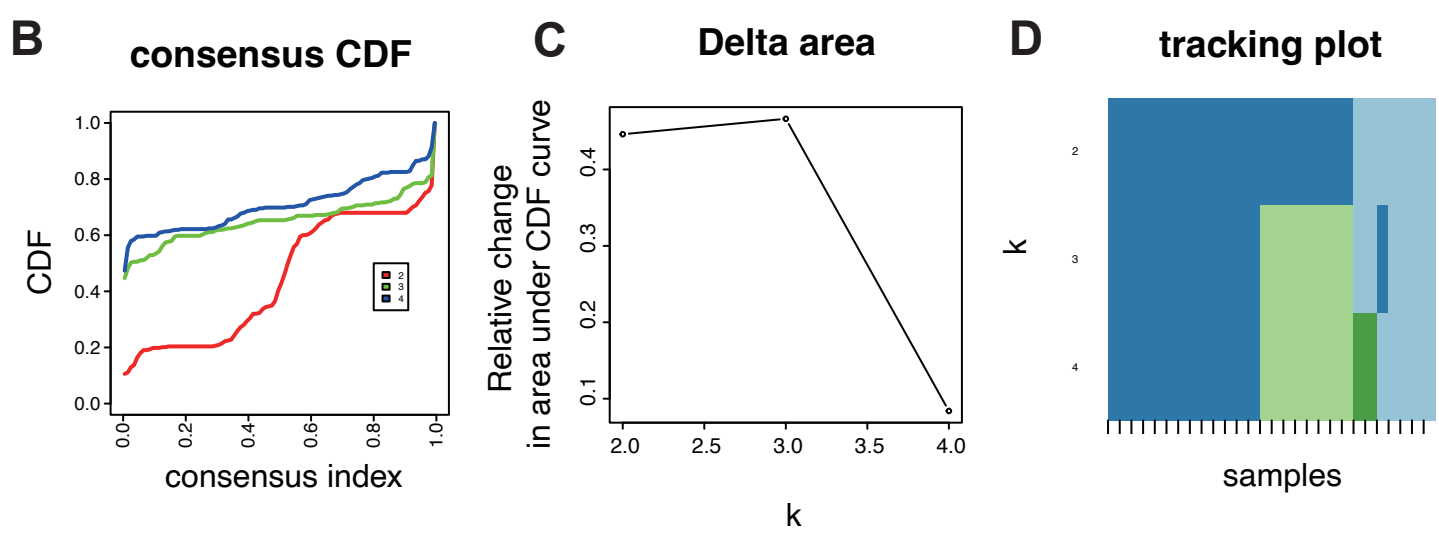

Supplement: Supplementary Figure 1 — Delineation of two distinct clusters through crotonylation profiling in glioma. (A) Consensus matrices across combined cohorts for each potential cluster number (k = 2–4), visualized through 1000 iterations of hierarchical clustering. (B) Cumulative Distribution Function (CDF) plot demonstrating the probability distribution based on consensus scores for each k in the combined cohorts. (C) Changes in the area under the CDF curve, expressed relatively. (D) Tracking plot illustrating variations in sample clustering for different k values. [file DataSheet1.pdf]

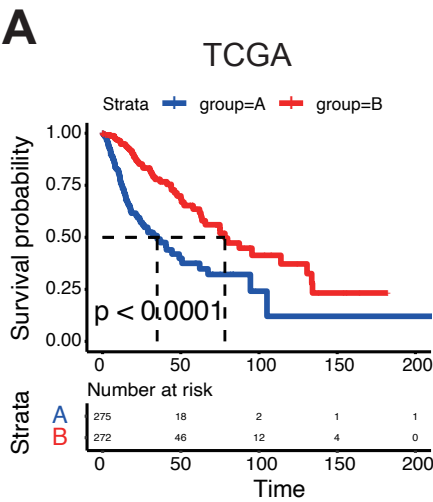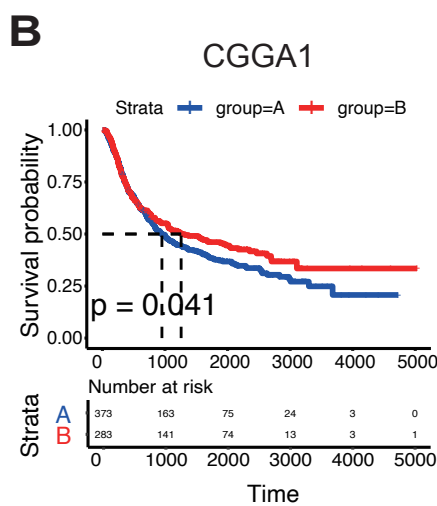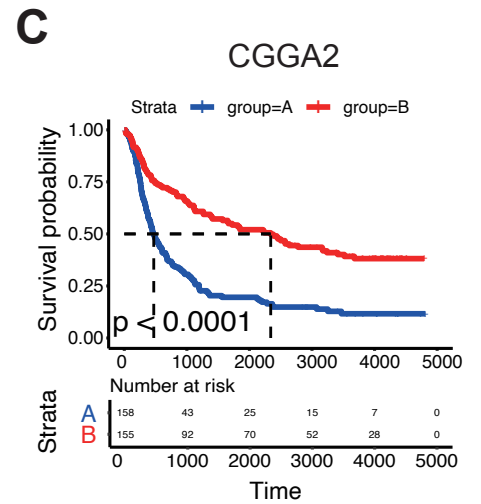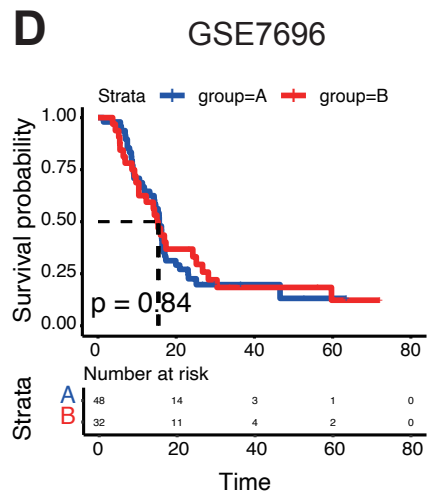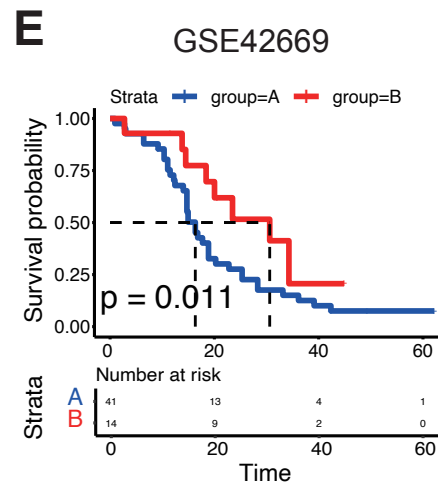

Supplement: Supplementary Figure 2 — Kaplan-Meier analysis for cluster A and cluster B generated after unsupervised clustering of crotonylation-related genes across five datasets. (A) TCGA, (B) CGGA1, (C) CGGA2, (D) GSE7696, (E) GSE42669. [file DataSheet2.pdf]

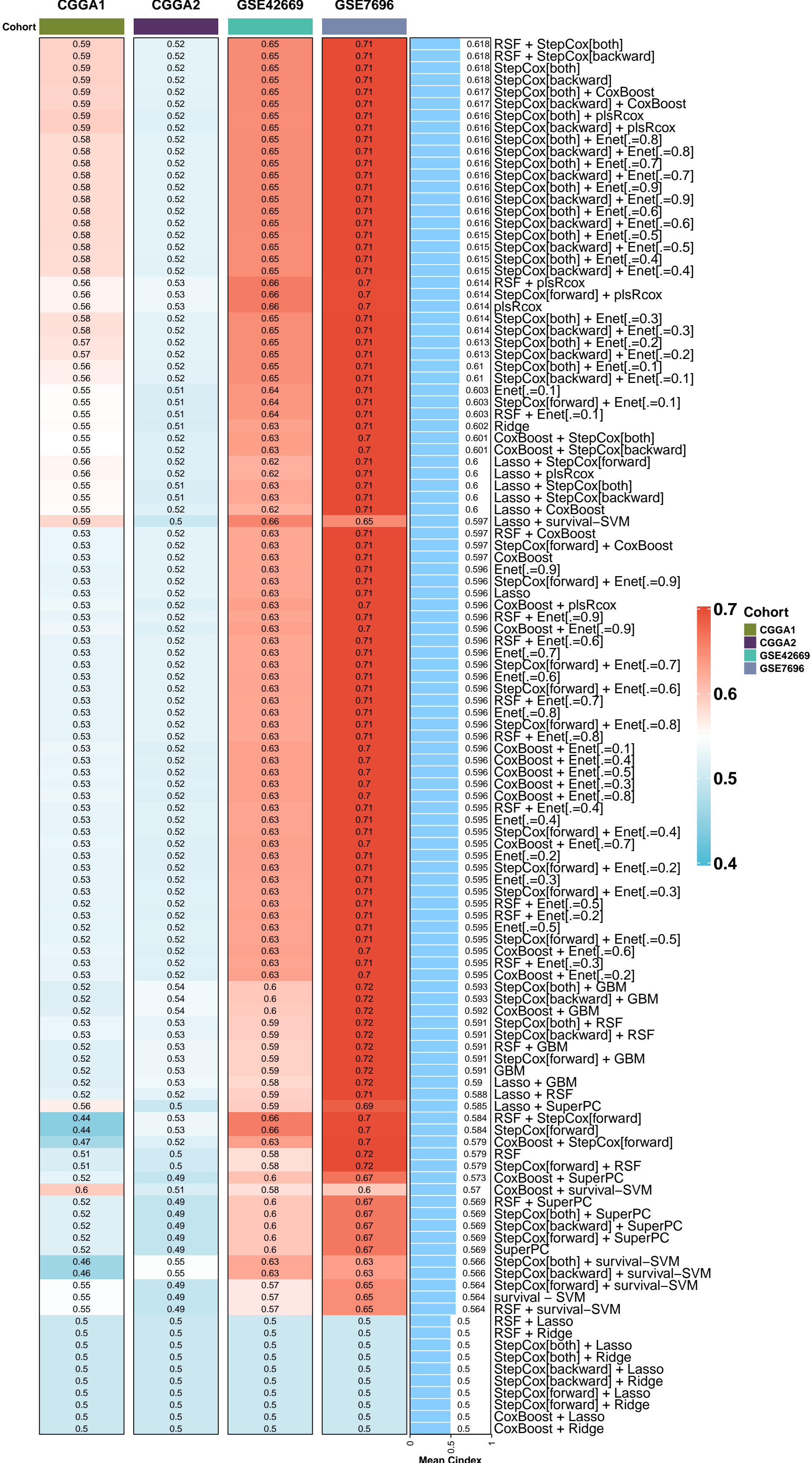

Supplement: Supplementary Figure 3 — Illustration of the machine learning-based integrative procedure. This figure demonstrates a comprehensive evaluation involving a total of 117 predictive models, with subsequent calculation of the Concordance Index for each model across all validation datasets. [file DataSheet3.pdf]

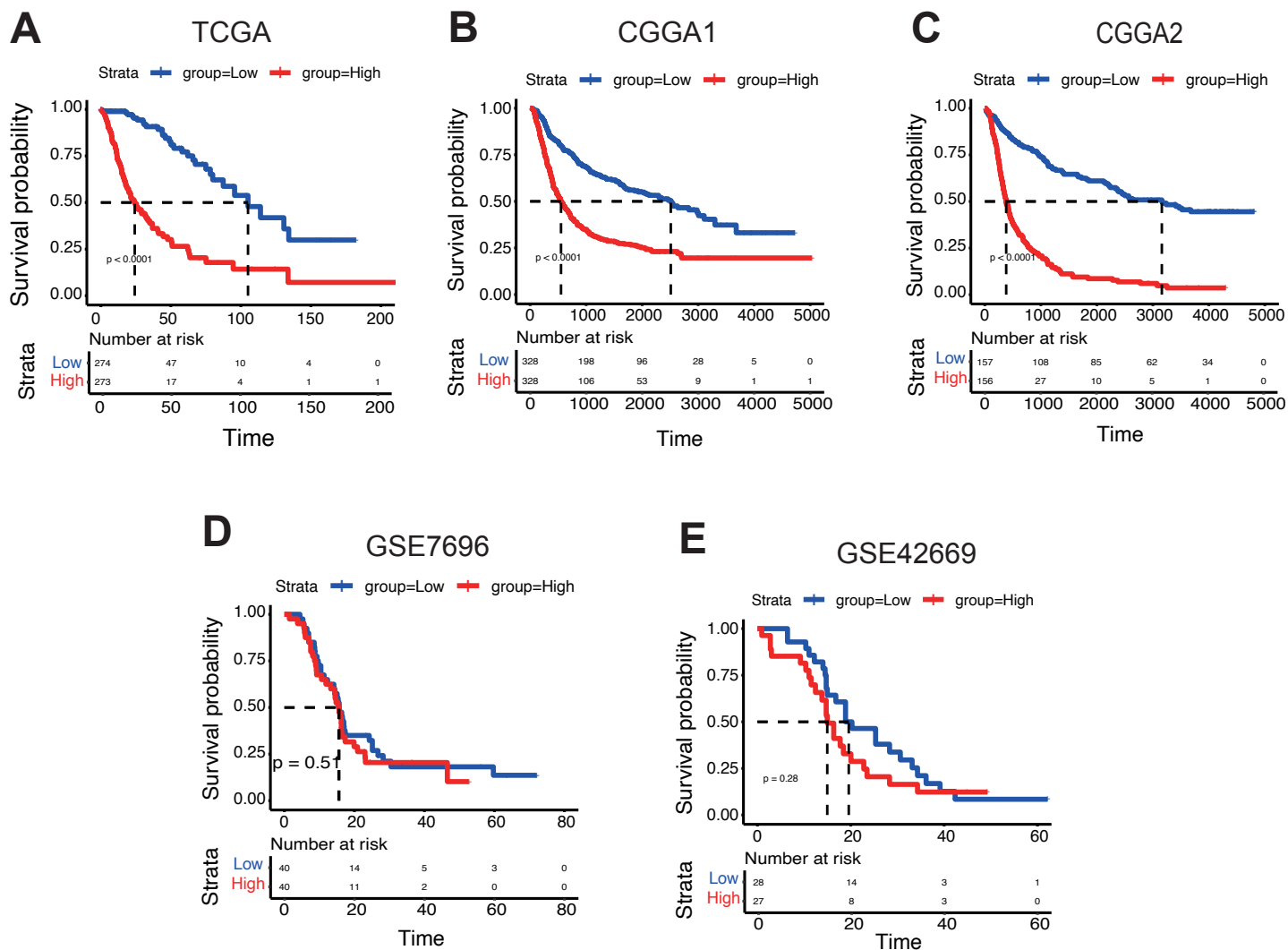

Supplement: Supplementary Figure 4 — Kaplan-Meier analysis for high- and low-risk groups across five datasets. (A) TCGA, (B) CGGA1, (C) CGGA2, (D) GSE7696, (E) GSE42669. [file DataSheet4.pdf]

**A**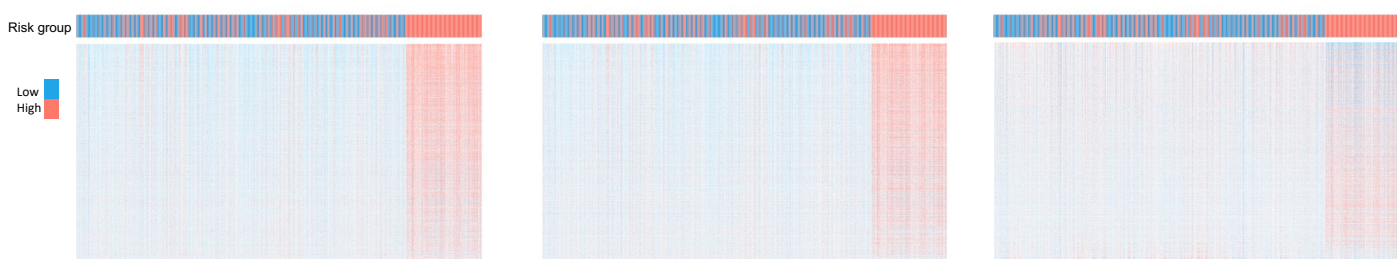**B**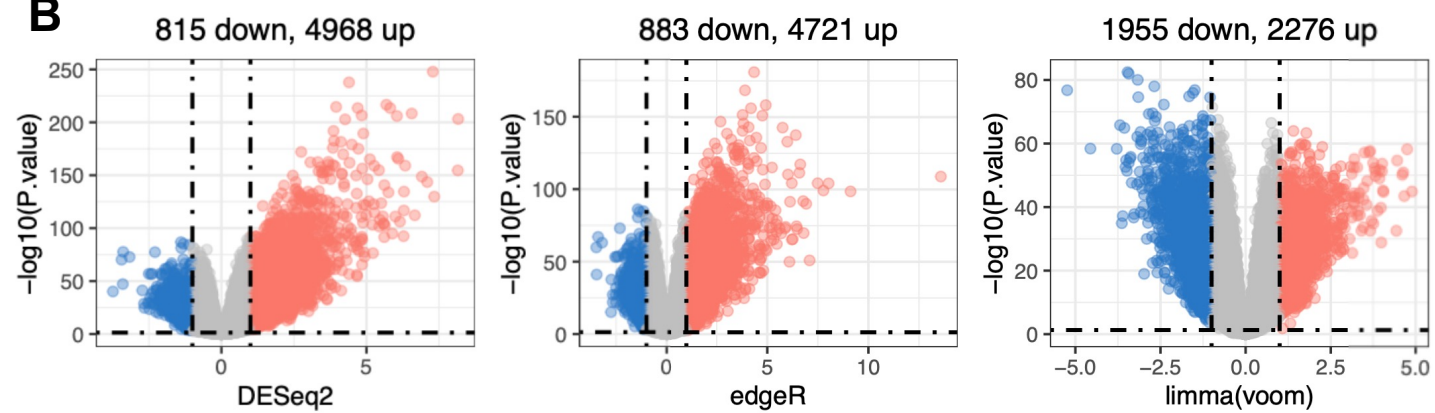

Supplement: Supplementary Figure 5 — Differential gene analysis between high-risk and low-risk samples in glioma. (A) Heatmaps displaying differential gene expression between high-risk and low-risk samples analyzed using the DESeq2 (left), edgeR (center), and limma (right) packages in R. (B) Volcano plots illustrating upregulated (red) and downregulated (blue) genes in high-risk versus low-risk samples, as identified by DESeq2 (left), edgeR (center), and limma (right) analyses. [file DataSheet5.pdf]

**A**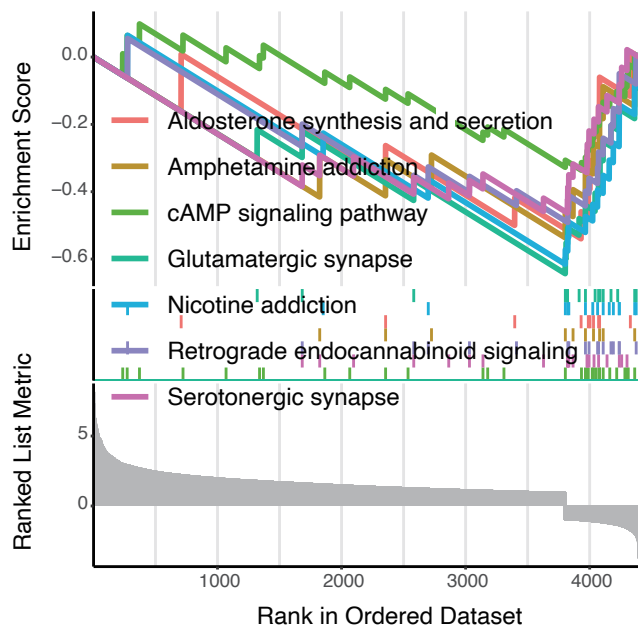**B**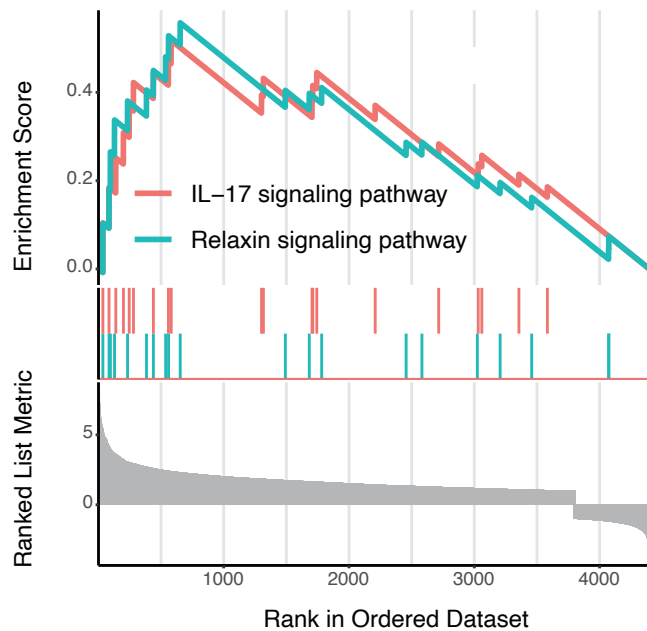**C**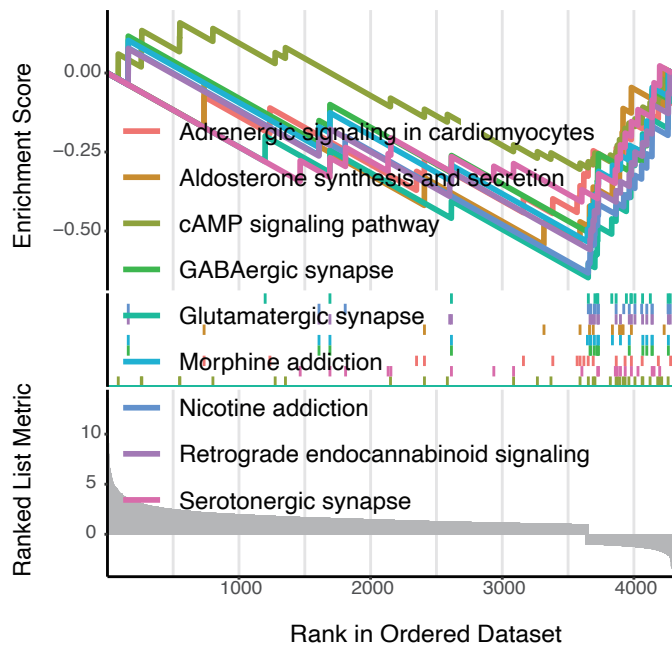**D**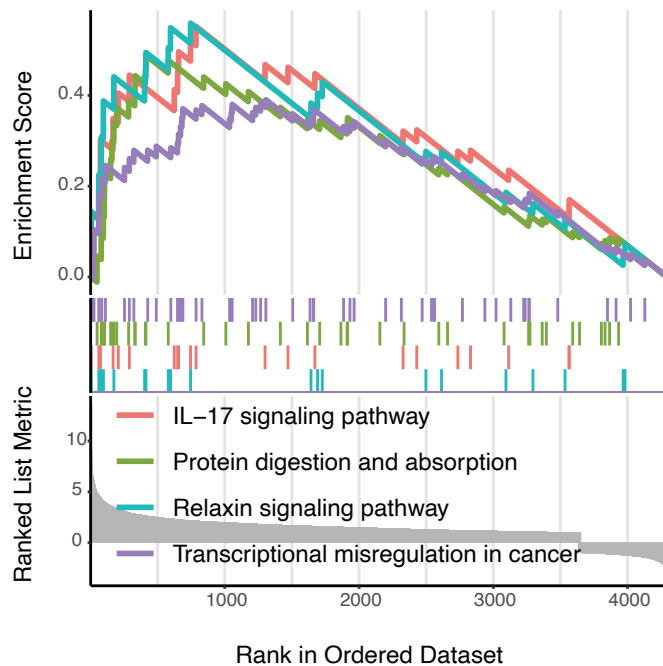

Supplement: Supplementary Figure 6 — Pathway enrichment analyses in low-risk and high-risk Groups. (A, B) GSEA analysis showing pathway enrichment of differentially expressed genes analyzed by the DESeq2 package in the low-risk group (A) and high-risk group (B). (C, D) GSEA analysis showing pathway enrichment of differentially expressed genes analyzed by the edgeR package in the low-risk group (C) and high-risk group (D). [file DataSheet6.pdf]

**A**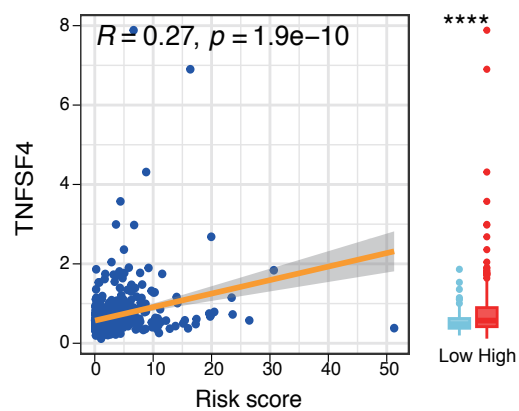**B**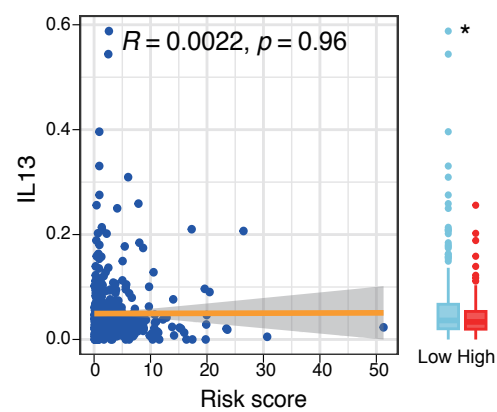**C**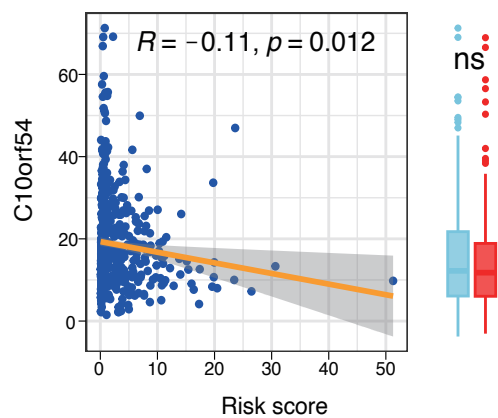**D**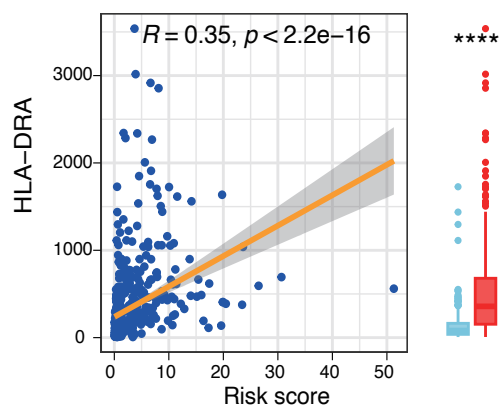**E**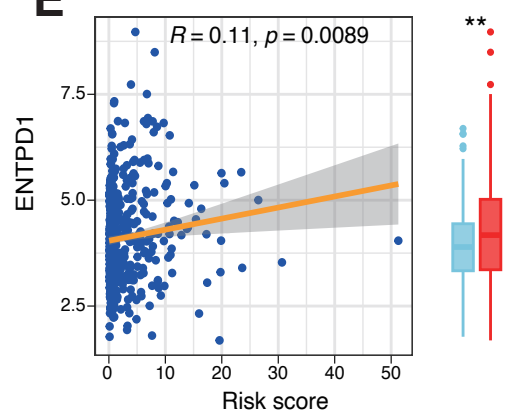

Supplement: Supplementary Figure 7 — Correlation analysis between the risk score and mRNA expression levels. (A) TNFSF4, (B) IL13, (C) C0orf54, (D) HLA-DRA, and (E) ENTPD1. [file DataSheet7.pdf]

**A**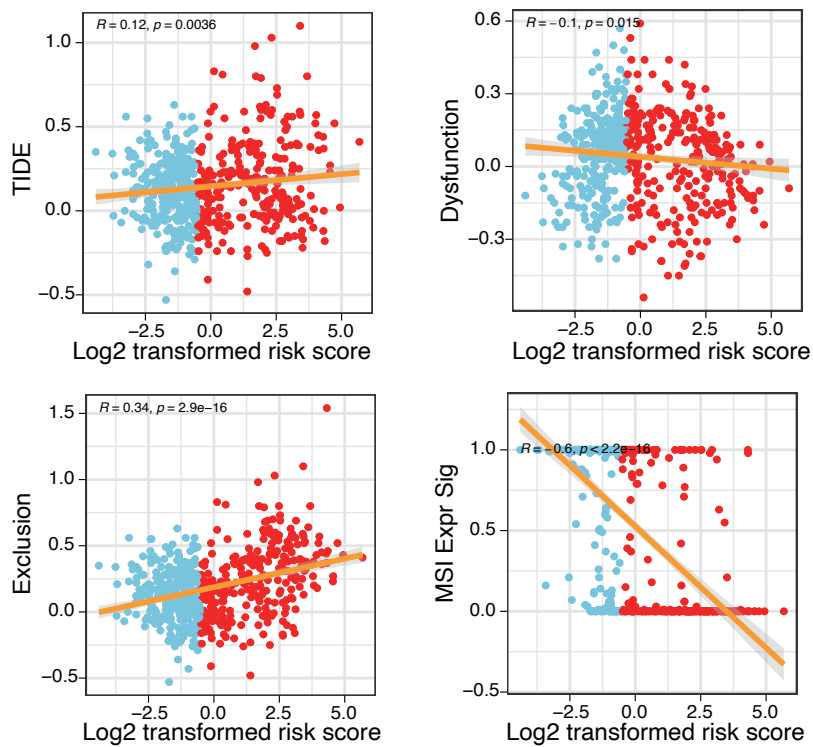**B**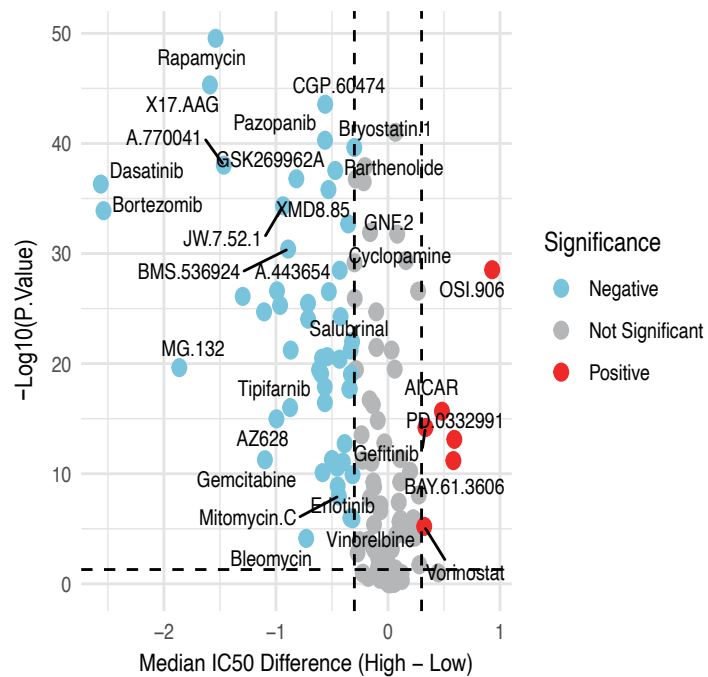**C**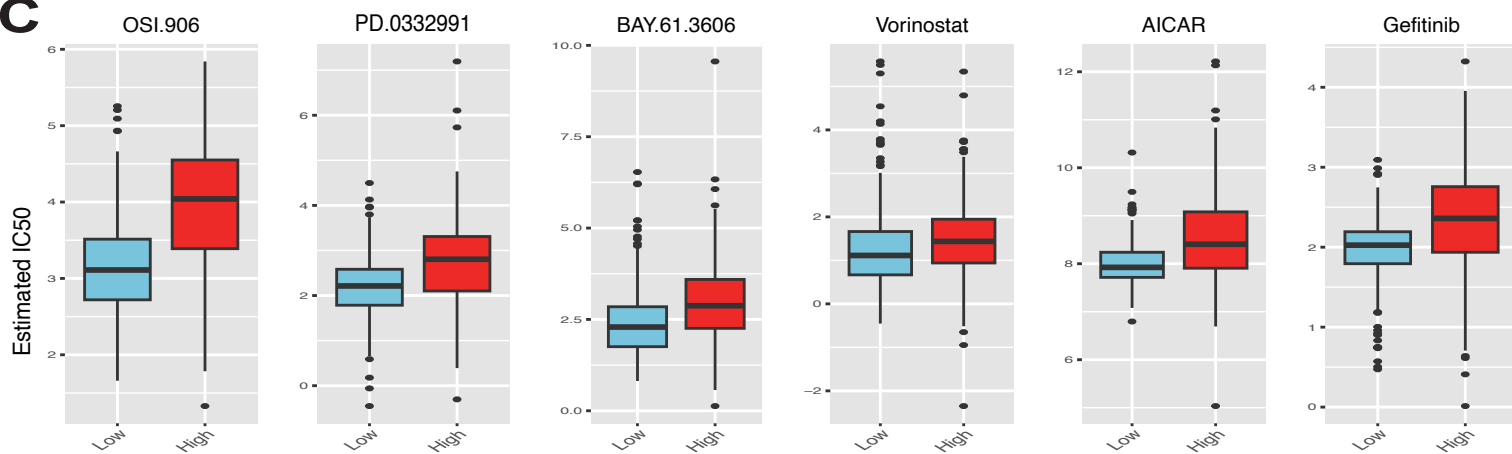**D**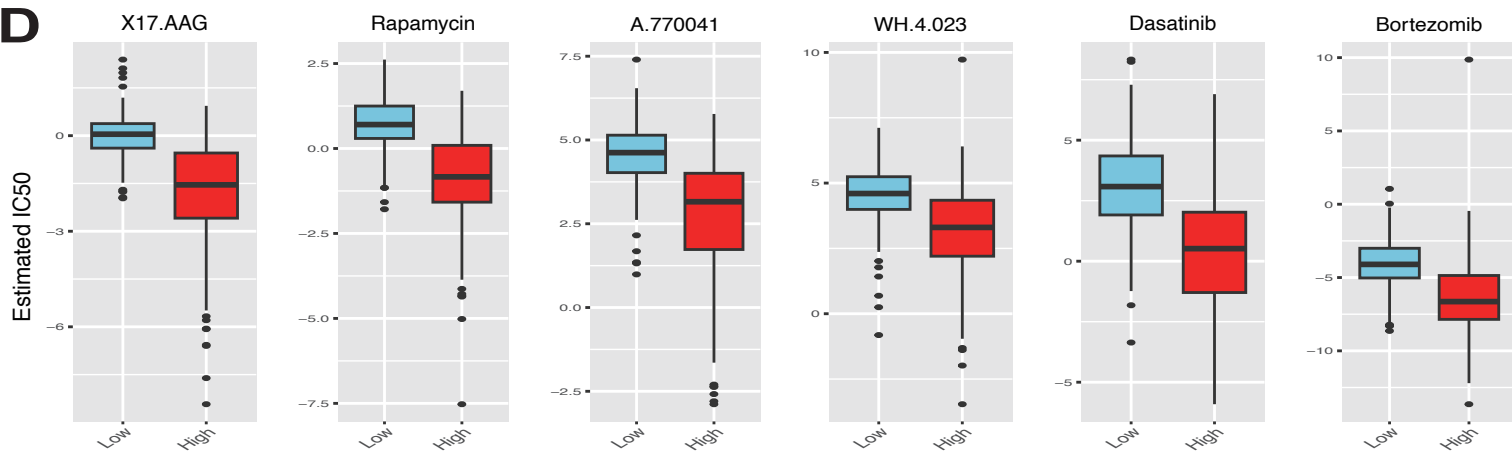

Supplement: Supplementary Figure 8 — Predictive value of the risk score in immunotherapy and chemotherapy. (A) The correlations between the risk score with TIDE score, dysfunction score, exclusion score, and MSI expression signature. (B) The relation between the IC50 of candidate drugs and risk scores. (C, D) Boxplots showing the estimated higher IC50 values of drugs in the high-risk group (C) and low-risk group (D). [file DataSheet8.pdf]

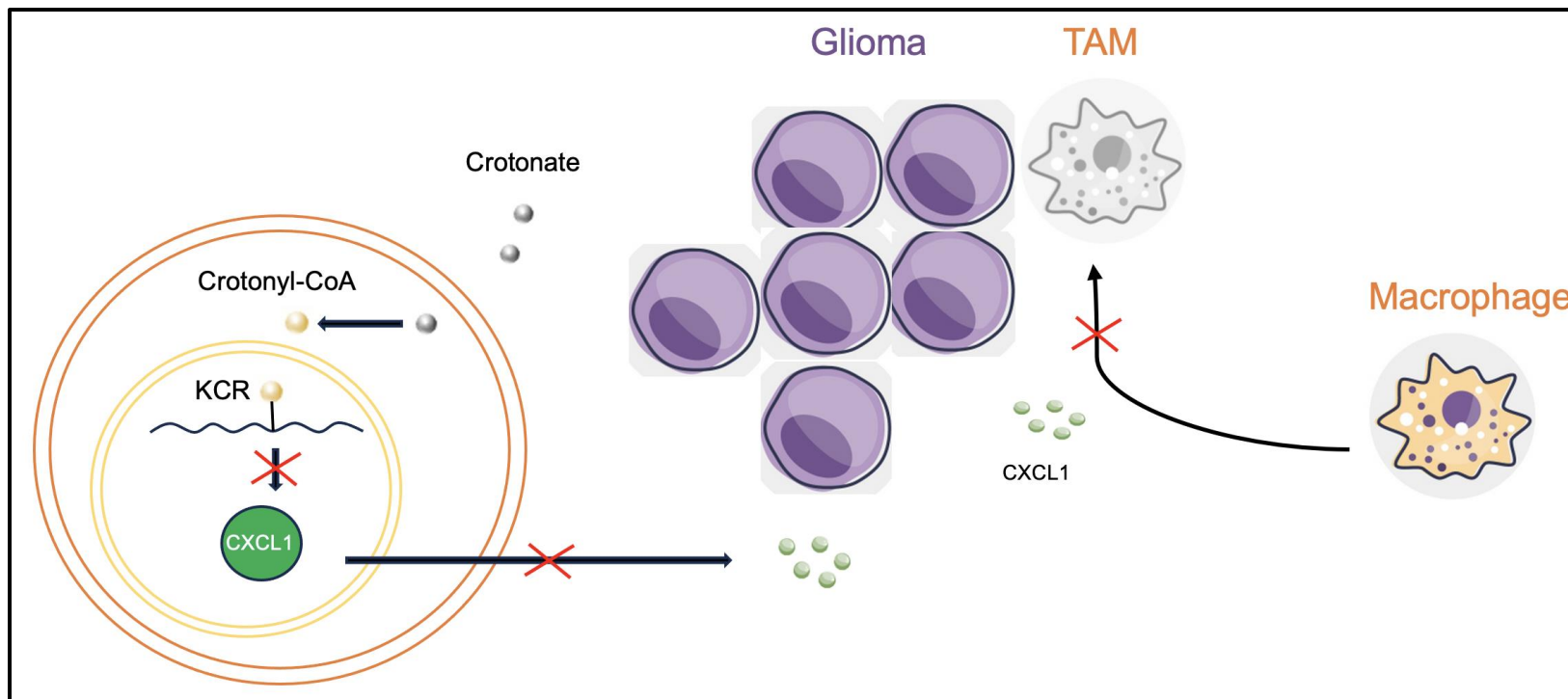

Supplement: Supplementary Figure 9 — Validation of SingleR cell type annotation accuracy using lineage-specific marker genes. [file DataSheet9.pdf]

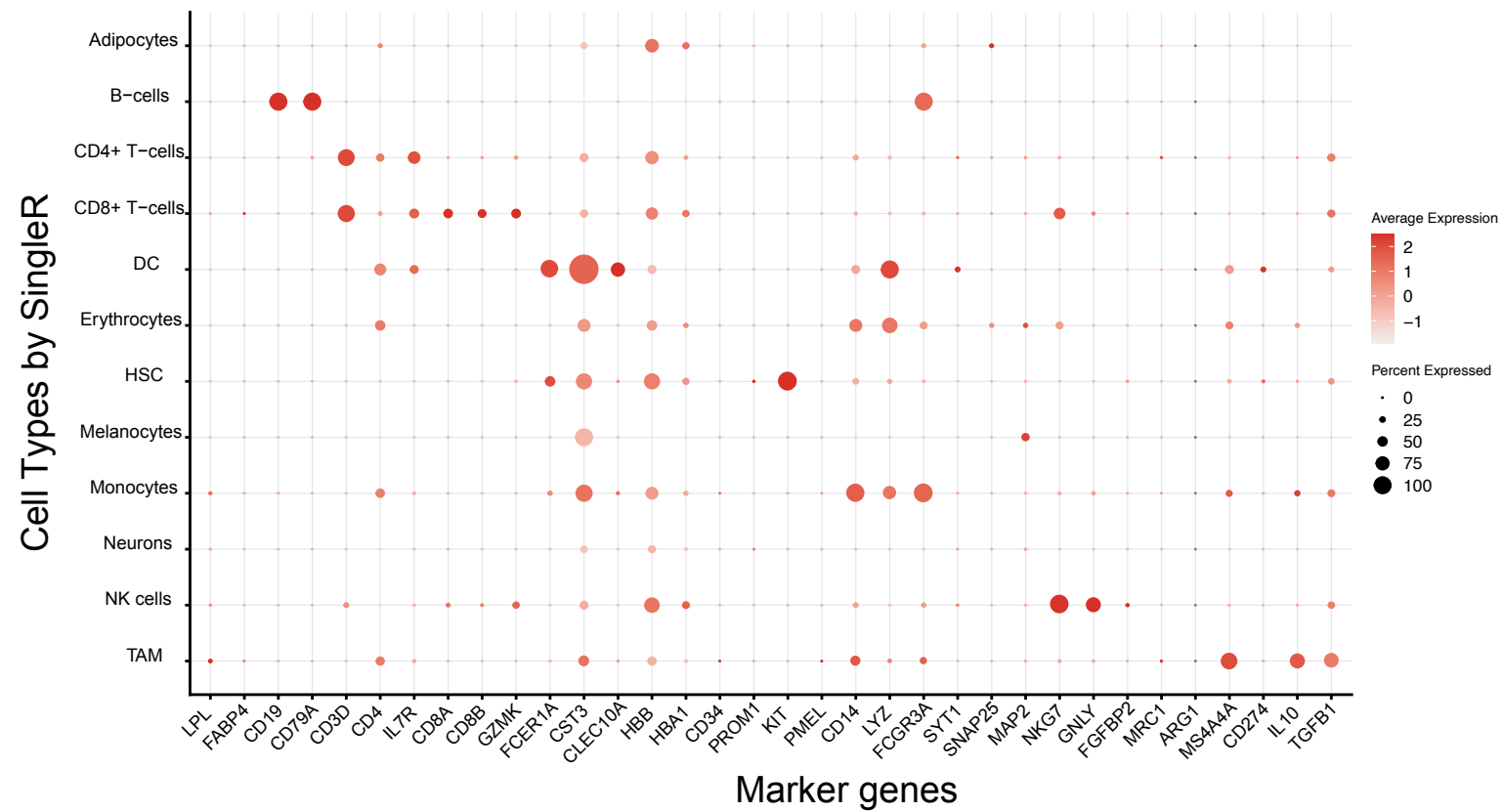

Supplement: Supplementary Figure 10 — Proposed mechanism of crotonylation-mediated regulation of macrophage infiltration and polarization in gliomas. Elevated intracellular crotonyl-CoA levels in glioma cells enhance global crotonylation, including histone crotonylation modifications. This epigenetic alteration induces chromatin structural remodeling, leading to transcriptional repression of the chemokine CXCL1. Reduced CXCL1 secretion into the tumor microenvironment diminishes monocyte recruitment via CXCR2 signaling, thereby suppressing tumor-associated macrophage (TAM) infiltration. [file DataSheet10.pdf]
